# Contribution of land use to the interannual variability of the land carbon cycle

**DOI:** 10.1038/s41467-020-16953-8

**Published:** 2020-06-23

**Authors:** Chao Yue, Philippe Ciais, Richard A. Houghton, Alexander A. Nassikas

**Affiliations:** 1 State Key Laboratory of Soil Erosion and Dryland Farming on the Loess Plateau, Northwest A&F University, Yangling, Shaanxi 712100 People’s Republic of China; 2 Laboratoire des Sciences du Climat et de l’Environnement, LSCE/IPSL, CEA-CNRS- UVSQ, Université Paris-Saclay, 91191 Gif-sur-Yvette, France; 3 Woods Hole Research Center, Falmouth, MA 02540 USA

**Keywords:** Biogeochemistry, Carbon cycle, Climate sciences, Carbon cycle, Carbon cycle

## Abstract

Understanding the driving mechanisms of the interannual variability (IAV) of the net land carbon balance (S_net_) is important to predict future climate–carbon cycle feedbacks. Past studies showed that the IAV of S_net_ was correlated with tropical climate variation and controlled by semiarid vegetation. But today’s land ecosystems are also under extensive human land use and management. Here, we report a previously hidden role of land use in driving the IAV of S_net_ by using an improved biosphere model. We found that managed land accounted for 30–45% of the IAV of S_net_ over 1959–2015, while the contribution of intact land is reduced by more than half compared with previous assessments of the global carbon budget. Given the importance of land use in modulating future land climate–carbon cycle feedbacks, climate mitigation efforts should strive to reduce land-use emissions and enhance the climate resilience of carbon sinks over managed land.

## Abstract (web summary)

Terrestrial carbon uptake as high inter-annual variability which can be used to help understand future responses to climate change. Here the authors’ modeling reveals a large portion of this variability is driven by human land use changes and management, and not captured by other models.

## Introduction

Annual growth rates of atmospheric CO_2_ show large interannual variations (IAV) that are dominated by changes in the net carbon balance of the land ecosystem^1^ (*S*_net_, with a positive sign indicating a carbon sink). The temperature sensitivity of such IAV provides us with clues of the strength of future land carbon uptake in response to global warming^2^. Advancing our understanding of the mechanisms controlling such sensitivity, including the climate sensitivity of *S*_net_, can help to reduce uncertainties of future projections by the coupled climate–carbon cycle models^3^. The IAV of *S*_net_ is linked to fluctuations in tropical land temperature and water storage, with higher sinks during cool and wet La Niña events, and lower sinks or even carbon sources during hot and dry El Niño events^4,5^. Recent studies have highlighted the role of semiarid biomes in dominating the IAV of *S*_net_ (refs. ^6,7^). However, to our knowledge, no studies have ever examined the role of human land use and management, in contrast to that of natural intact land, in driving the IAV of *S*_net_, despite the fact that land use and management exert increasing influences on the terrestrial carbon cycle^8^.

**Fig. 1:**
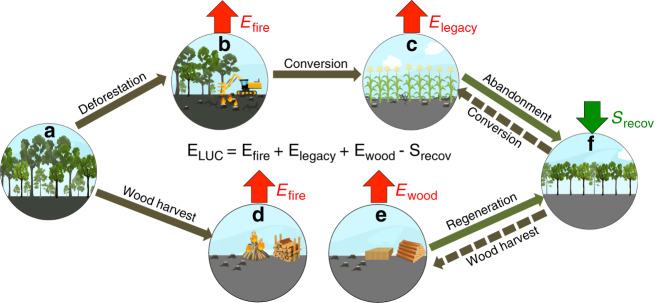
LUC processes and associated carbon fluxes. LUC processes considered in this study are: conversion of intact land (exemplified as intact forest (**a**)) into agricultural land (**c**), forest wood harvest for fuel wood (**d**), and industrial wood (**e**), and regeneration of secondary forest following harvest or agricultural abandonment (**f**). An old forest was used as an example for intact land in this figure, but similar land transitions involving natural grassland were also included. Likewise, pasture was also included as a form of agricultural land. The individual carbon fluxes comprising *E*_LUC_ are: **b**, **d** *E*_fire_, immediate emissions following forest clearing through burning of aboveground biomass and other on-site disturbance, plus emissions from harvested fuel wood assumed to be burned at the year of harvest; **e** *E*_legacy_, emissions from recently established agricultural land that is dominated by the decomposition of legacy slash and soil carbon inherited from former intact land; **b** *E*_wood_, long-term, gradual carbon release from industrial wood product degradation; and **f** *S*_recov_, carbon sink in recovering secondary forest and grassland. The net land-use change emissions (*E*_LUC_) is defined as: *E*_LUC_ = *E*_fire_ + *E*_wood_ + *E*_legacy_ − *S*_recov_, with a positive sign indicating a carbon source to the atmosphere. The dashed arrows indicate conversion of secondary forest (or grassland) into agricultural land in shifting cultivation, or reharvest of wood in case of forest management.

The land carbon balance can be separated into two additive components. The first one, land use and land-use change emissions (*E*_LUC_), denotes the carbon balance over lands under human land use, including agricultural land, managed forest, and secondary forest and grassland recovering from agricultural abandonment (for details of *E*_LUC_ components please refer to Fig. 1 and “Methods” section). *E*_LUC_ is an overall carbon source to the atmosphere because carbon emissions from land clearance generally outweigh sinks from afforestation and reforestation^9^. The second component carbon sink over intact land (*S*_Intact_) denotes the carbon balance over all lands that have remained intact from human perturbation since preindustrial times (defined as 1700) or have recovered to a similar status as intact ecosystems. Globally, *S*_Intact_ is a carbon sink driven mainly by environmental changes, including climate change, atmospheric CO_2_ growth, and nitrogen deposition^10^. In contrast, *E*_LUC_ is impacted by both direct human management actions, and environmental changes and variations.

The various *E*_LUC_ flux components, over a long term, are exposed to global environmental changes, and on yearly to multi-decadal scales, are subject to temporal variations driven by factors including climate variation and dynamics of human decisions leading to land-use conversion^11^. In the IPCC 5th Assessment Report (AR5)^12^ and the annual updates of the global carbon budget by the Global Carbon Project (GCP)^13^, *E*_LUC_ was calculated with bookkeeping models. Such models are forced with land-use reconstructions, and built upon fixed carbon densities and temporal response curves for different ecosystems following a land-use transition. Bookkeeping models excel in explicitly tracking land cohorts with different ages and resolving all *E*_LUC_ component fluxes, but any effects of environmental changes and climate variations are excluded. In IPCC AR5 and until recently in the GCP carbon budget^14^, *S*_Intact_ was thus quantified as a residual term of the global carbon balance (the bookkeeping and residual budget approach, see “Methods” section), absorbing all the potential biases in *E*_LUC_ estimated by bookkeeping models.

Bookkeeping models help to separate direct management and environmental effects in terrestrial carbon accounting^10^, but their results are not directly comparable with observations due to the static nature of the applied functions. Indeed, the lack of environmental effects on forest carbon densities in bookkeeping models has been argued as contributing to biases in forest carbon sink attribution^15^. Gridded dynamic global vegetation models (DGVMs) do include these effects and can account for land-use change (LUC), but most of them do not rigorously separate managed versus intact lands. Consequently, individual flux components of E_LUC_ cannot be resolved, making it impossible to reconcile the *E*_LUC_ estimates between DGVMs and bookkeeping models^16^. Moreover, recent studies highlighted serious confusion arising from such methodological inconsistency in quantifying and attributing anthropogenic carbon sinks. It has been proposed that the capabilities of DGVMs be expanded to represent sub-grid intact versus secondary lands to better represent the role of human land use and management in the land carbon cycle^17,18^.

In this study, we used a recently improved version of the ORCHIDEE DGVM, which is able to separate managed versus intact land at a sub-grid scale, to investigate the role of land use in modulating the IAV of *S*_net_. To highlight the difference of this improved DGVM and the bookkeeping approach in estimating *E*_LUC_ and its contribution to the IAV of *S*_net_, we implemented in ORCHIDEE the same LUC parameterization and forcing as one widely used bookkeeping model of Houghton and Nassikas^19^ (HN2017, see “Methods” section, Supplementary Note 1). For a baseline simulation to be consistent with the HN2017 study, we included only large-scale net LUC processes of deforestation, afforestation/reforestation, and transitions between natural grasslands and agricultural land, and wood harvest. We then further included local-scale shifting cultivation in a sensitivity simulation to explore the uncertainty of our results (see “Methods” section, Supplementary Note 2). The ORCHIDEE results were rigorously validated against various observations of deforestation area, forest biomass growth, global biomass distribution, and forest carbon sinks (see “Methods” section, Supplementary Note 3). For the period of 1850–2015, the temporal magnitude and changes of *E*_LUC_ derived from ORCHIDEE baseline simulation are in broad agreement with the HN2017 study, but the ORCHIDEE *E*_LUC_ shows much greater IAV. This suggests that human land use modifies the response of land ecosystems to climate variability and strongly modulates the IAV of *S*_net_. We found that managed land contributes 30–45% of the IAV of *S*_net_, in stark contrast to only 5% when *E*_LUC_ was derived by bookkeeping models.

## Results and discussion

### Spatial separation of *S*_net_ into managed and intact land

**Fig. 2:**
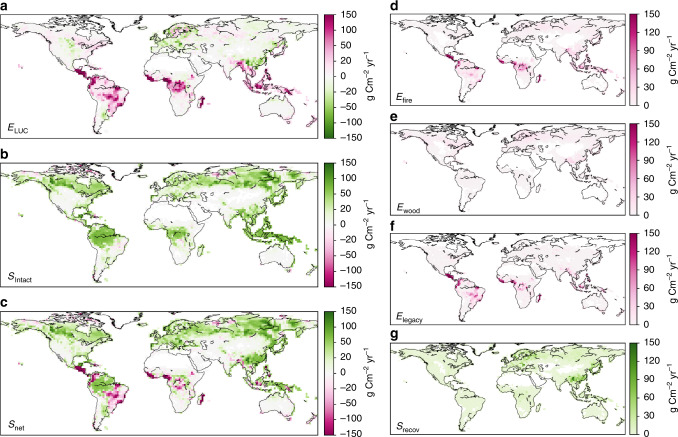
Land–atmosphere carbon fluxes over managed and intact lands. The annual mean values for 1990–2015 were shown. **a** *E*_LUC_ over managed land. **b** *S*_Intact_. **c** *S*_net_. The further disaggregation of *E*_LUC_ into its component emission fluxes of *E*_fire_, *E*_wood_, and *E*_legacy_, and the sink flux of *S*_recov_ are shown in **d**–**g**, respectively.

As is shown in Fig. 2, the ORCHIDEE model can rigorously separate carbon fluxes of managed and intact ecosystems (Fig. 2a–c, Supplementary Figs. 3 and 4). For the period of 1990–2015, *E*_LUC_ shows a net source of carbon in the tropics, driven by forest loss mainly due to the agricultural expansion, but less by industrial wood harvest^20^ (Fig. 2a). In contrast, over China, Europe, and part of the US, *E*_LUC_ is a net carbon sink as a result of forest management, afforestation, and agricultural abandonment^21,22^. *S*_Intact_ shows a spatially more uniform and diffuse sink of atmospheric CO_2_, driven by environmental changes (Fig. 2b). Consequently, the spatial pattern of *S*_net_ is largely dominated by *S*_Intact_, except for the region of arc of deforestation and the cerrado region in South America^20^, regions of central Africa, South Asia, and Southeast Asia, where deforestation-driven emissions outweigh intact land sink. Our estimated *E*_LUC_ and *S*_net_ for 1990–2015 were 1.54 Pg C year^−1^ and 1.06 Pg C year^−1^, respectively, within the range of *E*_LUC_ by HN2017 (1.23 ± 0.5 Pg C year^−1^) and consistent with the recent observation-based estimate of *S*_net_ by GCP using the residual approach (1.68 ± 0.8 Pg C year^−1^)^13^. The model validation points to an overestimation of forest biomass across the tropics by ORCHIDEE (Supplementary Fig. 6), which might lead to an overestimation of *E*_LUC_ from deforestation and consequently an underestimation of *S*_net_, partly explaining the lower simulated *S*_net_ compared to the observation-based estimate.

The spatial distribution of *E*_LUC_ was further disaggregated into its component fluxes as shown in Fig. 2d to Fig. 2g. The tropics are dominated by immediate emissions (*E*_fire_), and legacy slash and soil carbon emissions (*E*_legacy_), whereas emissions from wood product degradation (*E*_wood_) dominate the northern hemisphere, where forest management is widespread. Here movements of wood products by international trade were ignored. Carbon sinks from recovering secondary land are pervasive throughout the whole vegetated land, but more concentrated in Europe and eastern Asia. Our estimated global secondary forest carbon sink was 0.53 Pg C year^−1^ for 1990–2005 for a given secondary forest percentage of 19% to the total forest area, which was set to be consistent with HN2017 (Supplementary Note 4). A recent study estimated the global secondary forest sink as 1.30 (1.03–1.96) Pg C year^−1^ for 2001–2010 by assuming 61.4% of the total forest area^23^. Adjusting secondary forest to the same percentage in our study yielded a carbon sink of 1.69 Pg C year^−1^, being roughly consistent with the estimate of Pugh et al.^23^ (Supplementary Note 5). This carbon sink over secondary forest also compares favorably with the ~1.1 Pg C year^−1^ carbon sink over managed forests by including most countries of the world using forest inventory data, which were compiled recently by Grassi et al.^18^.

**Fig. 3:**
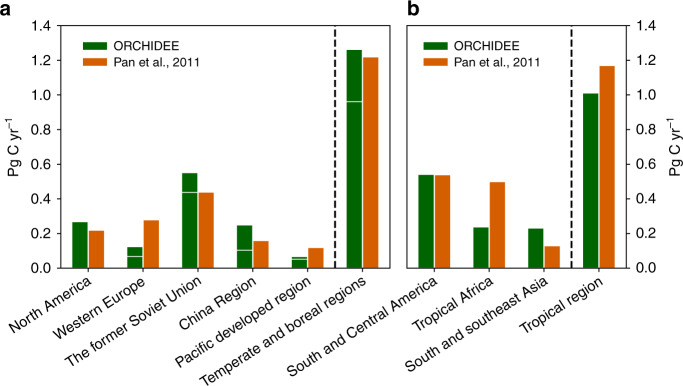
Forest carbon sinks by ORCHIDEE and an inventory-based study^24^. Data for 2000-2007 were shown. **a** Temperate and boreal regions, with sinks of intact (below horizontal white lines) and secondary (above horizontal white lines) forests being separated for ORCHIDEE simulation. **b** Forest carbon sinks over intact forests for tropical regions. Refer to Supplementary Fig. 1 for the global distribution of these eight regions.

We further compared carbon sinks from intact and secondary forests over different regions for 1990–2007, with a global synthesis of forest inventories^24^ (Fig. 3). For temperate and boreal regions, carbon sinks from intact and managed forests in ORCHIDEE were lumped together and compared with Pan et al.^24^ because distinctions were not made between these two forest types in the latter (Fig. 3a). The estimated forest sink over the whole temperate and boreal region by ORCHIDEE largely agrees with forest inventory data, being ~1.2 Pg C year^−1^ for 1990–2007, of which one fourth was contributed by managed forest. Managed forests have an especially large contribution in China and western Europe, highlighting the important role of forest management in these two regions. In North America, the carbon sink was completely dominated by intact forest while managed forest was carbon neutral, likely driven by large amounts of industrial wood harvest concurrent with a very low net forest gain according to the LUC forcing data used. Secondary forest carbon sink in tropical regions was estimated by Pan et al.^24^ using an earlier version of HN2017, but with great uncertainty in shifting cultivation. We therefore compared only intact forest sink with Pan et al.^24^ that was based on tropical forest plot data. Over the whole tropical region, the simulated intact forest sink was roughly comparable with the inventory data, being 1.0–1.2 Pg C year^−1^ for 1990–2007. ORCHIDEE underestimated the intact forest sink in tropical Africa, but overestimated the intact forest sink in South and Southeast Asia. The latter might be related to the overestimated forest aboveground biomass (Supplementary Fig. 6).

### IAV in *E*_LUC_ and its component fluxes

**Fig. 4:**
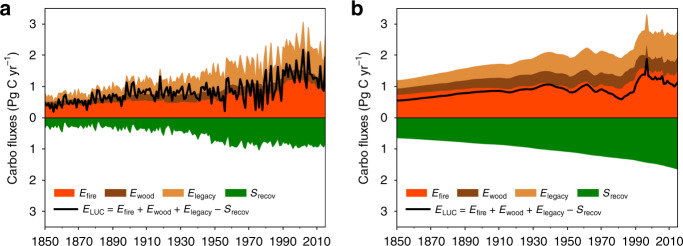
Comparison of *E*_LUC_ by ORCHIDEE and the bookkeeping model. **a** ORCHIDEE model. **b** The HN2017 bookkeeping model. Refer to Fig. 1 for the meanings of the four component fluxes of *E*_LUC_.

For the period of 1850–2015, our simulation results showed similar temporal patterns and magnitudes with HN2017, not only in the estimated global *E*_LUC_, but also in its individual components (Fig. 4). The main difference was the much higher IAV in *E*_LUC_ produced by ORCHIDEE. The IAVs of *E*_legacy_ and *S*_recov_ dominated the IAV of *E*_LUC_ in ORCHIDEE, in line with the model’s inherent capability to integrate ecophysiological impacts by climate variations. Our following analysis focuses mainly on the time period of 1959–2015, during which different components of global carbon budget can be relatively well constrained owing to reliable measurements of annual atmospheric CO_2_ growth rate^13^.

For 1959–2015, *E*_LUC_ estimated by HN2017 showed moderate variability with a variance of 0.07 Pg C year^−1^, with an apparent dominance by *E*_fire_, following land clearing and fuel wood harvest. By contrast, the variance of *E*_LUC_ estimated by ORCHIDEE was two times higher (0.20 Pg C year^−1^) and appeared to be dominated by *E*_legacy_ over agricultural land (with a variance of 0.09 Pg C year^−1^). This flux shows IAV mostly associated with the ENSO climate anomalies, with larger emissions during warmer and drier El Niño years, and lower ones during cooler and wetter La Niña years (Supplementary Fig. 8). This is because larger slash and soil carbon respiration coincides with reduced photosynthetic carbon uptake by cropland and pasture during El Niño years, whereas the reverse situation happens during La Niña years. This result is corroborated by eddy covariance flux measurements, suggesting a higher IAV in the net CO_2_ exchange of pasture and crop ecosystems than the forests they replaced^25,26^. Indeed, our model simulated much higher IAV in carbon fluxes on agricultural land than on intact forest or grassland (Supplementary Fig. 9). In contrast, for the results of the HN2017 bookkeeping model, the *E*_legacy_ flux mainly followed smooth decadal changes in land conversion with little interannual variability and no apparent association with climate variation (Supplementary Fig. 8). Note that the carbon balance of permanent agricultural land that existed before 1700 was also included in *E*_legacy_ in ORCHIDEE (see “Methods” section). However, their carbon balance was only a very small sink term of 0.06 ± 0.05 Pg C year^−1^ for 1959–2015, in contrast to a much larger *E*_legacy_ of 0.79 ± 0.31 Pg C year^−1^ over post-1700 agricultural lands. We therefore conclude that pre-1700 permanent agricultural lands have a negligible contribution to the magnitude, and IAV of *E*_legacy_ and *E*_LUC_ (Supplementary Fig. 10).

*E*_fire_ includes emissions from deforestation and fuel wood harvest. As forcing data for these processes were shared between ORCHIDEE and HN2017, it is reasonable that both *E*_fire_ estimates showed similar temporal patterns (Fig. 4). In reality, the dynamics of deforestation in the tropics are driven by both complex social and economic factors, as well as suitable climate conditions that allow effective removal of aboveground forest biomass^11,27^. The HN2017 result exhibited more IAV than ORCHIDEE in *E*_fire_ after 1995, because it included peatland fire emissions from an independent fire emissions database^19^, whereas this was not included in ORCHIDEE. This explains the fact that *E*_fire_ by HN2017 showed mild correlation with ENSO climate variations for 1959–2015 in Supplementary Fig. 8, while that by ORCHIDEE did not. In general, the IAV of *E*_fire_ was underestimated in both results, because the input deforestation areas were derived from FAO statistics at a five-year interval and therefore smoothed in time^19^. In addition, both methods used static coefficients to partition cleared biomass during deforestation into fire and other on-site disturbances. This approach dampens the IAV of deforestation fires emissions, because it does not account for climate-driven variations in combustion completeness, or potential time lags between the clearance and actual burning of forest biomass^28^.

In addition, ORCHIDEE and HN2017 both used as input data annually harvested fuel wood volumes from FAOSTAT during 1961–2015, and from compilations of historical information^19^. The harvested fuel wood showed low IAV perhaps due to only small variations in annual economic demand. For industrial wood, in ORCHIDEE carbon release was assumed evenly distributed over a product residence time (10 years and 100 years) and in HN2017 an exponential decay was assumed with the same residence times (10 years and 100 years). Both approaches approximated the slow, gradual destruction of industrial wood products and the return of their stored carbon into the atmosphere. These factors explain the low IAV in *E*_wood_ in both approaches.

Recovering secondary forest showed a long-term increase in carbon sink since 1850, both by ORCHIDEE and HN2017 (flux *S*_recov_ in Fig. 4), which reflected its growing area (Supplementary Fig. 4). But *S*_recov_ by ORCHIDEE showed larger IAV, which contributed to the IAV of *E*_LUC_, and had a relationship to ENSO that was in the antiphase to *E*_legacy_ (Supplementary Fig. 8). Recent analysis based on a satellite-derived proxy of plant photosynthesis pointed out that young forests tend to be more sensitive to precipitation variability than mature forests because of their shallower root systems, confirming the role of secondary forests in modulating the IAV of the land carbon balance^29^. Being roughly consistent with such empirical findings, ORCHIDEE also indicated higher IAV in *S*_recov_ than *S*_intact_ of intact forest mainly in the southern hemisphere (Supplementary Fig. 9). Supplementary Fig. 9 suggests that fluxes over managed land (*E*_legacy_ and *S*_recov_) have higher IAV than those over intact forest or grassland, demonstrating the contribution of land use to the IAV of *S*_net_.

Both gross emissions and recovery sinks estimated by HN2017 were higher than ORCHIDEE, and the difference was more pronounced when going further back in time (Fig. 4). This was likely because the response functions for forest recovery used in HN2017 are static and based on contemporary observations, with higher carbon stock and faster growth rates than actual historical values due to global environmental changes. In fact, the global secondary forest sink of 1.5–2 Pg C year^−1^ of HN2017 in 2000–2009 was higher than several other estimates. Using satellite-derived forest age distribution and the LPJ DGVM, Pugh et al.^23^ estimated a global secondary forest sink of only 0.53 Pg C year^−1^, excluding environmental change effects, much lower than HN2017. Similarly, the global secondary forest carbon sink estimated by Shevliakova et al.^30^ of 0.35–0.6 Pg C year^−1^ using the LM3V model, and the estimate by Yang et al.^31^ of 0.36 Pg C year^−1^ using the ISAM-NC model were both lower than HN2017.

**Fig. 5:**
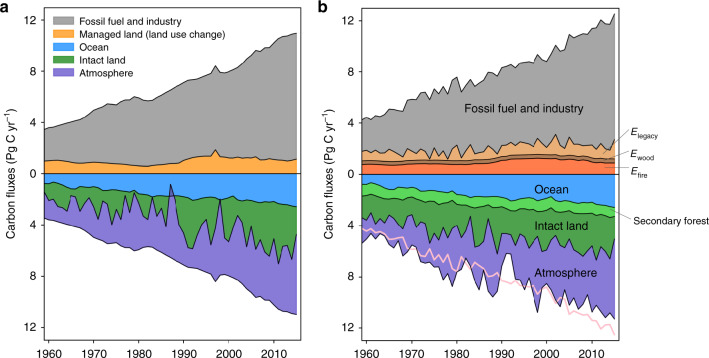
Overview of the global carbon cycle for 1959–2015. **a** *E*_LUC_ was derived from the HN2017 bookkeeping model with the intact land sink, as a residual of the global carbon budget. **b** All *E*_LUC_ component fluxes and the intact land sink were simulated by ORCHIDEE. Note that all land fluxes by ORCHIDEE include full impacts of environmental changes and climate variations. The difference between the bottom edge of the atmosphere sink and the pink line in **b** indicates the mismatch between the modeled and observation-based global carbon budgets.

Integrating different components of *E*_LUC_ along with their IAVs into the global carbon cycle yielded a different look for the global carbon budget than conventionally seen in IPCC AR5 and, until recently, in the annual carbon budgets released by the GCP (ref. ^14^; Fig. 5). The conventional picture shows managed land as a single, composite *E*_LUC_ with little IAV. *S*_intact_ was derived as the residual of other budget terms and it alone absorbs most of the IAV of *S*_net_, and drives almost completely the atmospheric CO_2_ growth variation (Fig. 5a). This dominance of IAV by *S*_intact_ remains unchanged in the most recent GCP global carbon budget^32^. The new picture following the ORCHIDEE simulation accounts for all the component source and sink terms over managed land, and disaggregates the whole gross land sink more realistically into secondary and intact ecosystems (Fig. 5b). Land-use-induced gross emissions (*E*_fire_, *E*_wood_, and *E*_legacy_) included both direct management effects and environmental effects, and exhibited greater IAV. As more IAV is attributed to managed land, intact ecosystems contribute less to the IAV of *S*_net_.

### Separating IAV in *S*_net_ into managed versus intact land

**Fig. 6:**
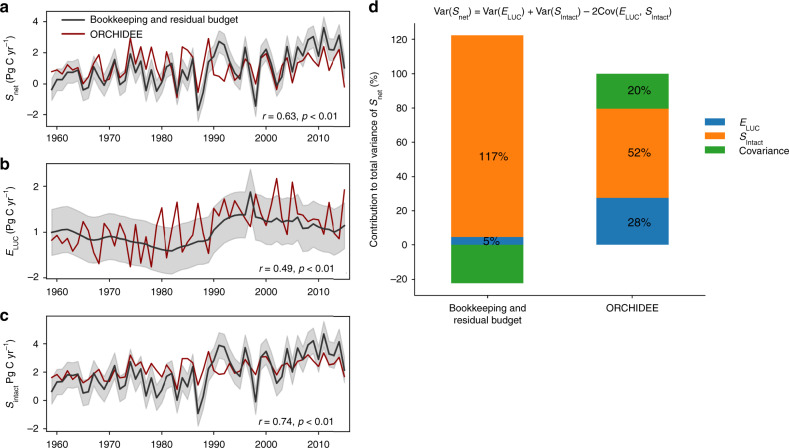
Attribution of *S*_net_ and its variance into *E*_LUC_ and *S*_Intact_. **a** *S*_net_. **b** *E*_LUC_. **c** *S*_Intact_. Data were shown for 1959–2015. The red line indicates ORCHIDEE result; the black line shows the result, following the bookkeeping and residual budget approach (i.e., IPCC AR5 and ref. ^14^), with shaded gray areas indicating the uncertainty of one standard deviation. Pearson correlation coefficients (*r*) between the two estimates are shown, with the period of 1991–1993 under strong Pinatubo influence being excluded when calculating *r* (*n* = 54, *p*-value calculated as a two-sided *p*-value). **d** shows the decomposition of temporal variances (Var) of *S*_net_ into the variances of *E*_LUC_, *S*_Intact_, and their covariance (*n* = 57, Eq. (5) in “Methods” section).

**Fig. 7:**
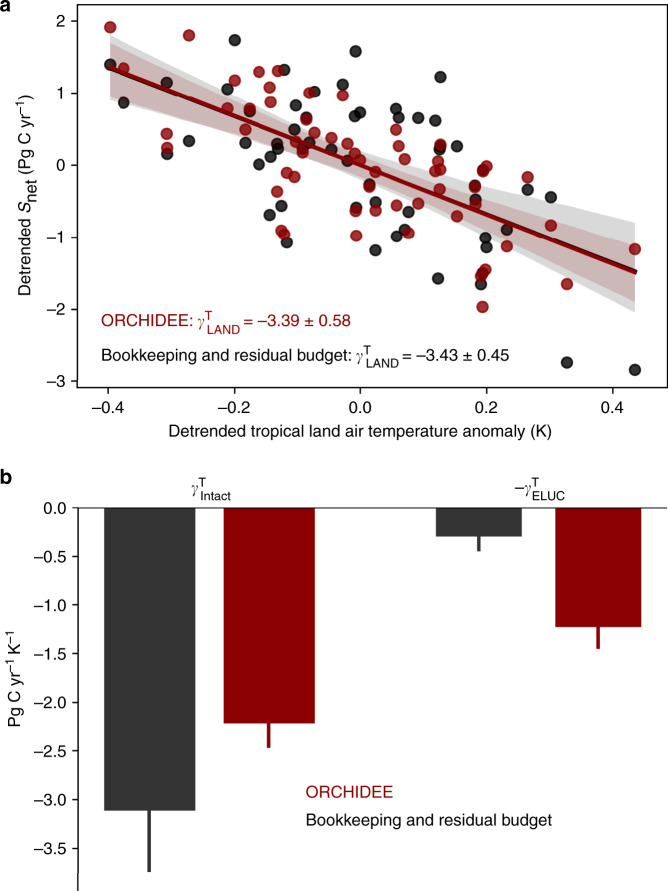
Temperature sensitivity (*γ*) of *S*_net_, *S*_Intact_, and *E*_LUC_. Red color indicates results from ORCHIDEE. Black color indicates results for the bookkeeping and residual budget approach (i.e., IPCC AR5 and ref. ^14^). **a** The temperature sensitivities of S_net_ ($\gamma _{\mathrm{LAND}}^{\rm{T}}$). **b** The decomposition of $\gamma _{\mathrm{LAND}}^{\rm{T}}$ into $\gamma _{\mathrm{Intact}}^{\rm{T}}$ and $- \gamma _{{\mathrm{ELUC}}}^{\rm{T}}$, following the equation “*S*_net_ = *S*_Intact_ − *E*_LUC_”. Negative values of $\gamma _{{\mathrm{LAND}}}^{\rm{T}}$ and $\gamma _{{\mathrm{Intact}}}^{\rm{T}}$ mean that elevated tropical land warming leads to less land carbon uptake, while positive values of $\gamma _{{\mathrm{ELUC}}}^{\rm{T}}$ mean that warming leads to enhanced carbon emissions from managed land (note that $- \gamma _{{\mathrm{ELUC}}}^{\rm{T}}$ is shown in the figure). All linear regressions were significant with a *p* < 0.05 (*n* = 57, two-sided *p*-value). Shaded area in subplot **a** indicates the 95% confidence interval of fitted values. Error bars in subplot **b** indicate the standard error of fitted *γ* values.

Figure 6 further compares in detail *S*_net_, *E*_LUC_, and *S*_intact_ derived using the bookkeeping and residual budget approach, and simulated by ORCHIDEE for 1959–2015. The *S*_net_ simulated by ORCHIDEE showed reasonable agreement with the observation-constrained value, with a Pearson correlation coefficient of 0.63 (*p* < 0.01; Fig. 6a). Additionally, *E*_LUC_ and *S*_Intact_ simulated by ORCHIDEE also agreed with the estimates from HN2017 and ref. ^14^ within their respective uncertainties (Fig. 6b, c), but it is clear that ORCHIDEE simulated a higher IAV for *E*_LUC_ with a lower IAV for *S*_intact_. Furthermore, the model also reproduced the observed sensitivity of *S*_net_ to tropical land temperature variations (Fig. 7a). This suggests that ORCHIDEE can adequately simulate *S*_net_ and its IAV (despite the underestimation of the variance of *S*_net_), giving us the confidence to further attribute the IAV of *S*_net_ into the components of *E*_LUC_ and *S*_Intact_, and to estimate their respective temperature sensitivities.

Figure 6d shows how the temporal variance of *S*_net_ was partitioned into the effects of *E*_LUC_ and *S*_Intact_, and their covariance term (Eq. (5) in “Methods” section). The ORCHIDEE results indicated a considerable *E*_LUC_ contribution of 28% of the global IAV in *S*_net_ compared with only 5%, when *E*_LUC_ was calculated with the HN2017 bookkeeping model. Conversely, intact ecosystems explained 52% of the variability of *S*_net_ in ORCHIDEE, whereas in the classical approach of bookkeeping and residual budget, *S*_Intact_ accounted for nearly all of the variability of *S*_net_. *E*_LUC_ and *S*_Intact_ following IPCC AR5 and ref. ^14^ had a positive covariance, but it is difficult to judge whether such a covariance is realistic or an artifact. For the ORCHIDEE results, *E*_LUC_ and *S*_Intact_ were calculated independently, and showed a negative covariance, which is consistent with the ecological responses to climate variation of the tropical land carbon cycle, i.e., that wetter climate conditions drive a larger-than-normal sink in both intact and managed lands, such that *E*_LUC_ is a smaller source during wet years when *S*_Intact_ is a larger sink (Supplementary Fig. 8). If we ascribe half of this covariance to the contribution by *E*_LUC_, then managed land contributes to ~40% of the IAV of *S*_net_.

Shifting cultivation mainly occurred in tropical regions (Supplementary Fig. 11), accounting for 2–4% of the total cropland area from 1500 to now. Given the local nature of shifting cultivation, we assumed that existing croplands were given a high priority to be put into fallow and most fallows were recycled within a certain rotation length (Supplementary Note 2). Taking a 20-year rotation length in the tropics^33,34^, accounting for shifting cultivation in ORCHIDEE (the SC-sensitivity run) yielded little new cropland being created after 1700, but generated 40% more secondary forests (Supplementary Fig. 12). As fallow secondary forests are constantly converted to cropland in shifting agriculture, simulated *E*_fire_ and *E*_legacy_ were higher in the SC-sensitivity run than in the baseline simulation. Despite an increase in *S*_recov_ consistent with the increase in secondary forest area, *E*_LUC_ remained higher in the SC-sensitivity run (Supplementary Fig. 13). This was particularly the case when shifting cultivation saw a rapid increase starting from 1950, when more intact forests were first cleared and then locked in the rotation cycle as secondary forests (Supplementary Fig. 11). The increase in *E*_LUC_ further decreased both *S*_net_ and *S*_intact_, but the IAVs of the three carbon fluxes remained similar to the baseline simulation (Supplementary Fig. 14). As a consequence of enhanced land use by including shifting cultivation, the contribution of land use to the IAV of *S*_net_ further increased to 45%, when half of the covariance term was also included.

The smaller IAV of the intact land sink shown here has implications for quantifying climate–carbon cycle feedbacks. The sensitivity of *S*_net_ to tropical temperature anomalies, defined as $\gamma _{{\mathrm{LAND}}}^{\rm{T}}$, was calculated using the ORCHIDEE baseline simulation and the observed *S*_net_ from the global carbon budget (see “Methods” section). Both methods yielded a $\gamma _{{\mathrm{LAND}}}^{\rm{T}}$ of –3.4 Pg C year^−1^ K^−1^ (Fig. 7a), but the temperature sensitivity of *S*_Intact_ ($\gamma _{{\mathrm{Intact}}}^{\rm{T}}$) by ORCHIDEE was only –2.2 Pg C year^−1^ K^−1^, in contrast to a $\gamma _{{\mathrm{{Intact}}}}^{\rm{T}}$ of –3.1 Pg C year ^−1^ K^−1^ when *S*_Intact_ was calculated as a residual of the global carbon budget using *E*_LUC_ from HN2017 (Fig. 7b). This suggests that excluding IAV in bookkeeping-derived *E*_LUC_ leads to a high bias in $\gamma _{{\mathrm{Intact}}}^{\rm{T}}$. On the other hand, managed land contributed to $\gamma _{{\mathrm{LAND}}}^{\rm{T}}$ with a larger role in controlling atmospheric CO_2_ variations than previously realized (Fig. 7b). The exact magnitude of its contribution is, however, subject to the setting of secondary forest proportion (19%, being consistent with the HN2017 model) in the present study. Accounting for shifting cultivation in the SC-sensitivity run slightly reduced $\gamma _{{\mathrm{Intact}}}^{\rm{T}}$ to a value of −2.0 Pg C year^−1^ K^−1^, with the relative role of managed land in driving $\gamma _{{\mathrm{LAND}}}^{\rm{T}}$ becoming even larger.

The ORCHIDEE simulation results presented here are subject to uncertainties. As discussed above, the IAV of *E*_fire_ and *E*_LUC_ were likely underestimated. On the other hand, cropland irrigation was not simulated, but it may affect the IAV of cropland carbon fluxes. Studies at county scale in the central US (ref. ^35^) and continental scale over Europe^36^ demonstrated that irrigation relieves water stress and the associated productivity drop over extreme drought periods. Irrigated croplands therefore may have a lower IAV in carbon uptake than rainfed croplands. Because 17% of global cropland was irrigated as of 2015 (ref. ^37^), neglecting irrigation processes in our model might lead to the overestimation of the IAV of *E*_LUC_.

Our study highlighted the role of land use in driving the IAV of the land carbon balance and its climate sensitivity. With historically ever-expanding areas subjected to human land use, humans today actively manage ~70% of the Earth’s total land surface^38^. A recent study reported a significant increase in the temperature sensitivity of the annual atmospheric CO_2_ increase over the past five decades^5^, but it remains unclear as to what extent expanding land use and management have contributed to such an increase. On the other hand, our results also call into question the reliability of using $\gamma _{{\mathrm{LAND}}}^{\rm{T}}$ as a single indicator to infer the future strength of climate–carbon cycle feedback, without accounting for the different sensitivities of carbon fluxes over intact versus managed lands, and the fact that structural changes in land allocation can influence $\gamma _{{\mathrm{LAND}}}^{\rm{T}}$. When it comes to inferring the future long-term sensitivity of managed land to climate change, management options such as planted forest species, forest rotation length, and the management of agricultural residues and soils are expected to strongly modulate the response of *E*_LUC_ to warming.

We suggest that to explain carbon cycle variations and to seek a better constraint on climate–carbon cycle feedbacks, more research attention should be directed toward the vast areas of managed land. Our results also highlight the importance of further expanding DGVM’s capability to individually separate managed and intact land, in order to evaluate the contributions of anthropogenic versus natural factors to the land carbon balance and its interannual variability. Future climate policy should be directed toward enhancing the climate resilience of forest sinks, and minimizing the legacy emissions from land use and management activities, for example, by selecting drought-resistant secondary forest species, extending the lifetime of woody products, and promoting carbon-retaining agricultural practices, such as no-tillage.

## Methods

### The bookkeeping and residual budget approach

In the IPCC AR5 (ref. ^12^) and the annual global carbon budget updates by the GCP (ref. ^13^), the *S*_net_ was derived using the following equation:

1 $${S}_{{\mathrm{net}}} = {E}_{{\mathrm{FUEL}}}-{S}_{{\mathrm{AIR}}}-{S}_{{\mathrm{OCEAN}}},$$

where *S*_net_ is the net land sink, *E*_FUEL_ is the CO_2_ emissions from fossil fuel burning and cement production, *S*_AIR_ is the atmospheric carbon sink in the form of CO_2_ growth for which reliable global measurements since 1959 are available, and *S*_OCEAN_ is the ocean carbon sink. All terms are defined as annual carbon fluxes. A positive sign of sink flux variables indicate removal of carbon from the atmosphere, while a positive sign of emission flux variables indicates release of carbon into the atmosphere. In this study, the fluxes of *E*_FUEL_, *S*_AIR_, and *S*_OCEAN_ for 1959–2015 were extracted from the Global Carbon Budget 2017 released by GCP (ref. ^13^).

We use the phrase net land sink for *S*_net_ because it is a net effect between two opposing terms: *S*_Intact_ subtracted by *E*_LUC_ from managed land:

2 $${{S}}_{{\mathrm{net}}} = {{S}}_{{\mathrm{Intact}}} - {{E}}_{{\mathrm{LUC}}},$$

where a positive sign of *E*_LUC_ indicates net release of carbon to the atmosphere. *E*_LUC_ occurs because relatively carbon-poor managed ecosystems replace carbon-rich intact ecosystems, and release their stored carbon into the atmosphere. *S*_Intact_ indicates carbon sink over land with no appreciable human modification and whose carbon sink can be mainly ascribed to global environmental changes, including atmospheric CO_2_ growth, climate change, and nitrogen deposition.

While *S*_net_ can be relatively well constrained following Eq. (1) with reliable estimates for all terms on the right hand side of the equation, neither *S*_Intact_ nor *E*_LUC_ can be directly measured over a large area; modeling therefore serves as the principal approach for their quantification. To estimate *E*_LUC_, bookkeeping models track over homogeneous geographical units the areas of forest loss and agricultural land gains, and subsequent abandonment, together with forest wood harvest and regrowth. Such land-use transition information is then further combined with carbon densities for various ecosystems, along with the temporal response curves of carbon pools after a land-use transition, to account for separately gross emissions, following land clearance and recovery sinks after agricultural abandonment and forest regrowth. In the IPCC AR5 and until the GCP annual carbon budget update for the year 2015 (ref. ^14^), *E*_LUC_ was predominantly estimated by the bookkeeping model of Houghton and colleagues^19,39^, which was widely adopted by the carbon cycle community. Subsequently, *S*_Intact_ is estimated by rearranging Eq. (2) as:

3 $${{S}}_{{\mathrm{Intact}}} = {{S}}_{{\mathrm{net}}} + {{E}}_{{\mathrm{LUC}}}.$$

In this approach, *S*_Intact_ is derived as a residual term of the carbon budget, and is often referred to as the residual land sink. In the current analysis, for carbon fluxes quantified using the bookkeeping and residual budget approach, i.e., following IPCC AR5 and ref. ^14^, we used the estimated *E*_LUC_ and its components from the most recent work by ref. ^19^ (hereafter shortened as HN2017), which includes large-scale historical deforestation and wood harvest (for details please refer to ref. ^19^) in land-use transitions. *S*_net_ and *S*_Intact_ were then calculated using Eqs. (1) and (3), respectively. Note that in the most recent global carbon budget by GCP (ref. ^32^), *S*_intact_ was derived by a group of DGVMs forced by constant preindustrial land cover distribution with varying atmospheric CO_2_, nitrogen deposition, and climate data. However, such *S*_Intact_ includes carbon fluxes over both managed and intact land today (rather than intact land only), and therefore the IAV of *S*_intact_ includes both managed and intact land as well, i.e., the separation of IAV in carbon fluxes between managed and intact land has not been done.

The HN2017 *E*_LUC_ estimate contains four different components according to the type of land-use transition involved and the time span over that the carbon flux occurs (main text Fig. 1): *E*_fire_ for immediate emissions following intact land clearance that often arise from burning of aboveground biomass residuals and other on-site disturbances, and are assumed to happen at the same year of clearance; *E*_wood_ for emissions from wood product degradation that extend over a long period after wood harvest; *E*_legacy_ for emissions over recently established agricultural land, resulting from the decomposition of slash and soil carbon as a legacy of former intact land; and *S*_recov_ for carbon sink in recovering secondary forest following agricultural abandonment or wood harvest. As such, *E*_LUC_ is quantified as:

4 $${{E}}_{{\mathrm{LUC}}} = {{E}}_{{\mathrm{fire}}} + {{E}}_{{\mathrm{wood}}} + {{E}}_{{\mathrm{legacy}}}-{{S}}_{{\mathrm{recov}}}.$$

The HN2017 bookkeeping model used fixed carbon densities and static temporal response curves of carbon stock change with time since land-use transition, and it was intended to include in *E*_LUC_ only the IAV due to changes in deforested area, but not those induced by climate variations and global environmental changes.

### Improved ORCHIDEE model with sub-grid land cohorts

Another approach to quantifying *E*_LUC_ is to use DGVMs that run over spatially explicit grids and numerically incorporate physiological vegetation carbon cycle processes, including photosynthesis, carbon allocation, vegetation mortality, and litter and soil carbon decomposition. In most DGVMs, areas of different vegetation or plant functional types (PFTs) are represented as separate tiles or patches in a model grid cell, over which carbon cycle, energy, and hydrological processes are simulated. In the majority of them only a single tile is used for a given PFT, and consequently, for instance, carbon fluxes of intact forest and recovering secondary forest cannot be distinguished^40^. This prevents them from estimating *E*_LUC_ by resolving each individual flux component in Eq. (4) as is done in bookkeeping models. Instead, for example, in most DGVMs that are used in the GCP annual carbon budget updates, *E*_LUC_ is derived from the difference between the *S*_net_ values in two parallel simulations: one with historical LUC and the other one without. Two features characterize such an approach: (1) compared with the *E*_LUC_ quantified using the bookkeeping method, *E*_LUC_ quantified by DGVMs includes the lost additional sink capacity that would otherwise occur in a world without any LUC but with atmospheric CO_2_ growth; and (2) in terms of the IAV in *E*_LUC_, IAV is dampened by subtracting carbon fluxes of two simulations that respond to climate variations in a symmetric way.

In this study, we used an improved version of the ORCHIDEE DGVM that is able to account for sub-grid cohorts for a given PFT that have different times since their establishment, so that the model has the strength to combine both bookkeeping functionality and the numerical representation of plant biophysics^40^. The ORCHIDEE version used here has been extensively validated for northern regions^41^ and applied globally in the recent annual GCP carbon budget update^13^. In this improved version, the carbon balances of intact and managed land (e.g., intact forest and recovering secondary forest) can be completely separated. This capability allows the quantification of *E*_LUC_ and its individual components following Eq. (4), but with the advantage of accounting for the full impacts of environmental changes on *E*_LUC_, and especially the impacts of climate variations.

Implementation of LUC. LUC processes shown in the main text Fig. 1 were implemented in ORCHIDEE in combination with the cohort functionality. For deforestation into agricultural land, intact forests were given a high priority to be cleared, reflecting the expansion of agricultural land in temperate regions over the history, and being consistent with the current-day agricultural expansion in the tropics. A certain fraction of aboveground biomass carbon was assumed as being released into the atmosphere within the same year as deforestation occurred, representing the common usage of fire for land clearing^33^. Unburned biomass residuals and root biomass carbon were transferred to litter pool of the new agricultural land, whose decomposition with time contributed to legacy emissions. When agricultural abandonment led to forest recovery, young secondary forest cohorts were established and further moved to older forest cohorts with their growth, until being declared again as intact when their biomass exceeded a certain threshold. Transitions between natural grassland and agricultural land were handled in a similar approach, except that all biomass carbon stocks were transferred to the litter pool (i.e., no fire-induced immediate carbon emissions), and higher priority was given to young cohorts in both conversions of grassland to agricultural land and agricultural abandonment into grassland. Due to a lack of savanna vegetation type in ORCHIDEE, land-use transitions involving savanna were integrated into those of forest and grassland. Because transitions between any pair of land-use types were explicitly represented in ORCHIDEE, the spatial-scale nature of LUC activities was independent of the spatial resolution of the model simulation, but depended on the input LUC forcing datasets (refer to Supplementary Note 6 for detailed discussions).

For both industrial and fuel wood harvest, we started from intact forests and then move to younger cohorts in order to fulfill the prescribed annual-harvested wood biomass in the forcing data. This is consistent with the approach used in HN2017. For fuel wood harvest, the aboveground woody biomass carbon was assumed being emitted into the atmosphere during the same year as harvest occurred, whereas small branches and leaves were moved to litter pool. In the case of industrial wood harvest, certain fractions of aboveground woody biomass were stored as two wood product pools with a 10- and 100-year residence time, respectively, while the unharvested branches and leaves, and root biomass were moved to litter pool. Following harvest, young forest cohorts were planted and underwent the same process as secondary forests generated by agricultural abandonment.

Simulation setup. Comparing *E*_LUC_ and its components estimated by ORCHIDEE and by HN2017, provided that they were driven by shared LUC reconstructions and follow the same LUC parameterizations, allows us to elucidate the IAV of *E*_LUC_ and its contribution to the IAV of *S*_net_. We first performed a baseline ORCHIDEE simulation to include the same LUC processes as in HN2017. These include large-scale processes, such as deforestation, afforestation/reforestation, and transitions between natural grassland and agricultural land, and wood harvest. For both ORCHIDEE and HN2017 estimates, the simulations were started from the year 1700, but the *E*_LUC_ was examined for the period of 1850–2015. The ORCHIDEE baseline simulation was driven by variable atmospheric CO_2_ and CRUNCEP climate data at a 2-degree resolution (prior to 1901 climate data for 1901–1920 were recycled). Historical forest area changes and wood harvest biomass were driven by exactly the same data used in HN2017 for different geographical regions of the world (see Supplementary Figs. 1 and 2; more details are provided in Supplementary Note 1). Gridded annual agricultural area changes were derived from the LUH2 dataset^42^. When agricultural area changes could not be matched by changes in forest imposed by HN2017, they were implemented as transitions with natural grassland. To ensure the comparability with the HN2017 bookkeeping model, we implemented the same LUC parameterizations in ORCHIDEE. Please refer to Supplementary Table 1 for details in association with various *E*_LUC_ flux components.

Shifting cultivation is a local-scale subsistence agricultural practice that involves conversion of forest or natural grassland into agricultural land, maintaining such agricultural land for a certain period, and then setting it as fallow and repeating the whole cycle. Despite its existence as an early form of human land use and being active in certain regions of the tropics today, the areas subjected to shifting cultivation were of great uncertainty^43^. Several DGVMs reported additional carbon emissions by further accounting for shifting cultivation, but its exact contribution to the global land carbon balance remains elusive^44^. For these reasons, shifting cultivation was not included in the HN2017 study, or the ORCHIDEE baseline simulation for consistency purpose. Nevertheless, for the purpose of uncertainty analysis of our baseline results, we performed an additional sensitivity simulation accounting for shifting cultivation starting as early as 1500 (the SC-sensitivity run). Historical areas subjected to shifting cultivation between forest (or grassland) and agricultural lands were extracted from the LUH2 data^42^ (see Supplementary Note 2 for details of shifting cultivation implementation in ORCHIDEE), while all other drivers were the same as the baseline simulation.

To balance between computing resources demand and accurate representation of land management status, six age cohorts were used for forest PFT and two age cohorts were used for herbaceous PFT (i.e., grassland, cropland, and pasture) in the ORCHIDEE simulation. The results indicated that intact forest and grassland, permanent agricultural land (pasture and cropland) existing prior to 1700, and agricultural land established after 1700 (post-1700 agricultural land), as well as recovering secondary forest and grassland were well separated throughout the simulation (Supplementary Figs. 3 and 4). Two herbaceous PFT cohorts represented permanent and post-1700 agricultural land, or intact and recovering grassland, respectively. The management status of forest related to either disturbance history or recovery status. Before the start of the simulation, all forests were considered as intact in the baseline simulation (the uncertainty of this assumption was tested in the SC-sensitivity run). As time evolved, forest age structure changed (Supplementary Fig. 4). As a first approximation, we considered old-growth secondary forests as intact forests, when their wood mass exceeded 90% of the maximum attainable wood mass determined under preindustrial conditions. This roughly corresponded to a forest age of 70, 90, and 160 years for tropical, temperate, and boreal forests, respectively. Such age thresholds were consistent with the reported secondary forest ages that reached a similar status as intact forests from field investigations^45^. To maximize the consistency with the HN2017 study, we further adjusted the composition of global intact and secondary forests, according to the HN2017 bookkeeping model at the end of the baseline simulation (i.e., 81% primary versus 19% secondary forests for the year of 2015, see Supplementary Note 4).

Model validation. The ORCHIDEE model was rigorously validated against observations to ensure a correct estimate of *E*_LUC_ in this study (for details see Supplementary Note 3). To properly simulate deforestation emissions, the spatial distribution of modeled aboveground biomass and deforestation area were compared with satellite observations (Supplementary Figs. 5 and 6). To capture secondary forest recovery, a database of forest biomass growth was constructed based on chronosequence observations. Modeled biomass–age relationships were then evaluated against this database for different forest types (Supplementary Fig. 7, Supplementary Table 2). Different from bookkeeping models, ORCHIDEE DGVM can account for global environmental changes and simulated forest carbon sinks can thus be compared with regional forest inventory data (Fig. 3). A top-down estimate of *S*_net_ could be reliably derived as a residual of carbon emissions minus sinks by the atmosphere and the ocean, both of which were based on observations. The magnitude and IAV of the simulated *S*_net_, and its sensitivity to tropical land temperature variation were compared with the observation-based *S*_net_ derived by the residual approach (Figs. 6a, 7a).

Posttreatment of ORCHIDEE simulation: The *S*_net_ is defined as the simulated net biome production (NBP) over the globe, and is equal to net primary production after subtracting heterotrophic respiration, fire CO_2_ emissions, and agricultural harvest. A positive NBP indicates carbon absorption by land. The NBP over intact forest and grassland was quantified as *S*_Intact_. For the *E*_LUC_ flux components, *E*_fire_ and *E*_wood_ in Eq. (4) can be easily identified in ORCHIDEE. We treated NBP over secondary forest and grassland (being net primary production less heterotrophic respiration and fire carbon emissions) as *S*_recov_, and the opposite of NBP over agricultural lands (croplands and pastures) as *E*_legacy_. We assumed that NBP over recently established agricultural land was dominated by the decomposition of legacy slash and soil carbon, originating from former intact land. Note that the *E*_legacy_ in ORCHIDEE also included carbon fluxes over permanent agricultural land existing prior to 1700. Its scope was slightly different from the bookkeeping approach, where only post-1700 agricultural lands were included. But such a definition was coherent with our research objective to investigate the role of all lands under human land use in driving the IAV of *S*_net_. Because permanent agricultural land showed only a small sink with very low IAV, its influence on the magnitude and IAV of *E*_LUC_ was negligible (Supplementary Fig. 10).

### Attribution of IAV in *S*_net_

*S*_net_ shows large IAV with temperature sensitivity providing a constraint on the magnitude of future climate–carbon cycle feedbacks. Following Eq. (2), to examine the IAV of *S*_net_ and attribute it to the effects of managed versus intact land, the temporal variance of *S*_net_ (Var (*S*_net_)) was decomposed into the variances of *E*_LUC_, *S*_Intact_, and their covariance (main text Fig. 6d):

5 $${\mathrm{Var}}\left( {{{S}}_{{\mathrm{net}}}} \right) = {\mathrm{Var}}\left( {{{E}}_{{\mathrm{LUC}}}} \right) + {\mathrm{Var}}\left( {{{S}}_{{\mathrm{Intact}}}} \right)-{\mathrm{2Cov}}\left( {{{E}}_{{\mathrm{LUC}}}{\mathrm{,}}\,{{S}}_{{\mathrm{Intact}}}} \right).$$

We also calculated the tropical temperature sensitivity of *S*_net_ ($\gamma _{{\mathrm{LAND}}}^{\rm{T}}$), *S*_Intact_ ($\gamma _{{\mathrm{Intact}}}^{\rm{T}}$), and *E*_LUC_ ($\gamma _{{\mathrm{ELUC}}}^{\rm{T}}$) as a way to infer climate–carbon cycle feedbacks (main text Fig. 7) for various land carbon fluxes. Tropical land air temperature anomalies were derived from the CRUNCEP climate data that forced the ORCHIDEE simulation, after removing the linear trend. Carbon flux anomalies were regressed against temperate anomalies using the ordinary least-squares linear regression, with the *γ* values given by the regression slopes.

## Supplementary information

Supplementary Information

Peer Review File

Supplementary Data 1

## Data Availability

The Global Carbon Budget 2017 data released by the GCP is available at https://www.icos-cp.eu/global-carbon-budget-2017. The LUH2 data are available at https://luh.umd.edu/data.shtml. The Source data underlying Figs. 2–7 are provided as a Source data file. The satellite-based tree cover loss data used in Supplementary Fig. 5 are available at https://earthenginepartners.appspot.com/science-2013-global-forest/download_v1.2.html. The availability of satellite-based biomass datasets used in Supplementary Fig. 6 is as follows: GEOCARBON: https://www.wur.nl/en/Research-Results/Chair-groups/Environmental-Sciences/Laboratory-of-Geo-information-Science-and-Remote-Sensing/Research/Integrated-land-monitoring/Forest_Biomass.htm; GlobBiomass: https://doi.pangaea.de/10.1594/PANGAEA.894711. CCI Biomass: http://cci.esa.int/biomass; Liu et al.^46^: http://wald.anu.edu.au/data_services/data/global-above-ground-biomass-carbon-v1-0/. Raw model output data and other data underlying all figures, including Supplementary ones, are provided at Figshare (10.6084/m9.figshare.12401615). The python scripts used to process the data are available from the corresponding author upon reasonable request.

## Code availability

The ORCHIDEE model code used in this study is open-source and distributed under the CeCILL (CEA CNRS INRIA Logiciel Libre) license. The code is hosted on the official ORCHIDEE svn server and tagged as revision 6545. It can be downloaded in a linux system using the svn command of “svn co --username anonymous svn://forge.ipsl.jussieu.fr/orchidee/branches/publications/ORCHIDEE_GLUC_r6545 ORCHIDEE”, with both username and password as “anonymous”. Guidance to install and run the model is provided at https://forge.ipsl.jussieu.fr/orchidee/wiki/Documentation/UserGuide. Access to the official run environment is restricted to registered users; requests can be sent to the corresponding author.
