# Supplementary material for: Contribution of land use to the interannual variability of the land carbon cycle
Source: Nat Commun. 2020 Jun 23;11:3170. doi: 10.1038/s41467-020-16953-8 (PMC7311403; doi:10.1038/s41467-020-16953-8)
Supplement: Supplementary file 1 — Supplementary Information [file 41467_2020_16953_MOESM1_ESM.pdf]

Supplementary Information

Contribution of land use to the interannual variability of the land carbon cycle

Yue et al

This file includes:

Supplementary Note 1 to Supplementary Note 6

Supplementary Figure 1 to Supplementary Figure 14

Supplementary Tables 1, 2

Supplementary References

Supplementary Note 1 Integrating historical forest area changes and wood harvest biomass used in HN2017 into ORCHIDEE

To ensure the consistency in land-use change (LUC) drivers between the ORCHIDEE simulation and HN2017 study, we integrated the historical forest area changes and wood harvest biomass used in HN2017 into ORCHIDEE based on geographical regions of the globe (Supplementary Figure 1 and Supplementary Figure 2). ORCHIDEE is a gridded model that needs spatially explicit LUC input data, but LUC drivers in HN2017 are provided on a regional basis. We therefore further used gridded LUC transition information from the 'Land-Use Harmonization' LUH2 data[1] (http://luh.umd.edu/, the v2h version) to allocate the total regional forest area change onto the level of model grid cells. The LUH2 land-use transition reconstructions were used in the annual global carbon budget updates and have been widely adopted by DGVM communities[2].

The gridded agricultural land area changes used in ORCHIDEE were constrained by those in the LUH2 data, which further used historical agricultural land information from the HYDE3.2 data set[3]. When backcasting historical land-use maps using the dual constraints of forest area change from HN2017 and agricultural land change from LUH2, the mismatches between the changes of these two land-use types were assumed being compensated by area changes in natural grassland. LUH2 data spatially maps the transitions of forested land into agricultural land, and we used such information to allocate the regional total forest area change onto each model grid cell. This approach ensured that while regionally the forest area change used in ORCHIDEE was exactly the same as in HN2017, the spatial distribution of deforestation and afforestation/reforestation conformed to the LUH2 data because the latter used a dedicated complex spatial modeling approach to guarantee its validity.

Supplementary Note 2 The SC-sensitivity simulation accounting for shifting cultivation from the year of 1500

The implementation of major land use change processes with the cohort functionality in ORCHIDEE provides the opportunity to simulate shifting cultivation with a certain rotation length. Shifting cultivation represents the slash-and-burn local-scale subsistence agriculture that involves conversion of forest or natural grassland into agricultural land, maintaining such agricultural land for a certain period and then setting it as fallow and repeating the whole cycle. Shifting cultivation was not included in HN2017 and our baseline simulation, mainly because of the great uncertainty in the magnitude and changes of its area over history[5]. Nonetheless, the impact of shifting cultivation on land carbon cycle has received increasing attention[7]. Here, we extracted simultaneous transitions with equal quantity between forest and cropland, as well as natural grassland and cropland from the gridded LUH2 data as cultivation shifted. The LUH2 dataset is the most recent up-to-date data of this kind to be used in DGVMs and ESMs in the upcoming IPCC 6th Assessment Report[1] (https://luh.umd.edu/data.shtml).

The LUH2 data provides land use transitions among forested land, non-forested land, cropland, managed pasture, and rangeland. We took the non-forested land as natural grassland and the total area of managed pasture and rangeland as pasture area. In LUH1, which was used in IPCC AR5 and was the predecessor of LUH2, shifting cultivation exists for both cropland and pasture[8]. But simultaneous equal land transitions were only found for transitions involving cropland in LUH2. The areas of shifting cultivation involving cropland in LUH2, however, remained close to those in LUH1.

Historical changes of areas subject to shifting cultivation, and their spatial distribution were shown in Supplementary Figure 11.

We searched through the literature to find information regarding the rotation length of shifting cultivation. Mertz et al.[9] reported fallow length mostly less than 30 years in Malaysia and Indonesia. Koch et al.[10] reported that fallow length in South America from as short as 4 years and as long as 30 years with an average of 15 years in Amazonia. The LUH1 data set also assumed a constant fallow length of 15 years[11]. Considering these study results, we decided to start clearing of forest from the intermediately-aged forest cohorts (Age 3 in the model), corresponding to a fallow length of 20 and 28 years in tropical and temperate regions, respectively. If intermediately aged cohorts were exhausted, the model then searched for older forest cohorts for shifting cultivation purpose. For the conversion of natural grassland into cropland in shifting cultivation, priority was given to the younger cohorts followed by the older ones. This is a reasonable assumption because shifting cultivation usually happens at a small spatial scale and subsistence farmers are limited in their ability to move, and as a result lands within a relatively small distance tend to be re-used in fallow cycles.

Note that the inclusion of shifting cultivation was made on top of the large-scale net land use change and wood harvest that were already included in the baseline simulation. All parameterizations related to land transitions in shifting cultivation followed the same as for net land use changes. Please refer to Supplementary Table 1 for parameterization details.

Supplementary Note 3 Model evaluation and comparisons with observations

Deforestation emissions (E_{fire} and E_{legacy}) largely depend on the biomass density of the forests to be cleared. Therefore, it is important for the model to represent realistically the spatial distribution of deforestation area in relation to biomass distribution, as well as the spatial distribution of forest biomass. In addition, to correctly simulate forest recovery (S_{recov}), the model must be able to capture the temporal patterns of forest biomass growth with age. We evaluated the forest loss distribution along biomass gradients against the medium-resolution satellite-based gross tree cover loss for 2000-2014. As is shown in Supplementary Figure 5, for the four regions where forest loss was dominated by net deforestation, the distribution of forest loss along the biomass density gradients in ORCHIDEE was in agreement with that from satellite observation by ref[4]. The forest loss at the lower end of the biomass density gradients, in particular of <20 Mg C ha^{-1}, was nonetheless underestimated in ORCHIDEE and LUH2 compared with the satellite observation, but its influence on estimated E_{LUC} was expected to be small because of the low carbon stock involved. In contrast, the slight overestimated deforestation areas in high-biomass areas might lead to an overestimation of deforestation carbon emissions in the model.

We further compiled a site-level chronosequence-based forest biomass growth database from the literature to validate forest recovery as simulated by ORCHIDEE (Supplementary Figure 7, Supplementary Table 2). The database contained 1093 observations of forest aboveground biomass from 131 sites covering tropical, temperate, and boreal regions with distinctions being made between broadleaf and coniferous forests. The forest biomass growth simulated by ORCHIDEE for tropical raingreen forest largely followed the observed pattern, despite the overestimation for the late successional stages (Supplementary Figure 7b). The overestimation was more pronounced when observations were compared with the simulated growth by tropical evergreen broadleaf forest. This might indicate a high bias in the simulated tropical forest biomass. The model can realistically

simulate forest growth for temperate and boreal forests except for temperate coniferous forests (Supplementary Fig. 4c to 4e), where the model showed strong underestimation mainly because the highly productive evergreen coniferous forests in western US and coniferous plantations in eastern Asia showed extremely high aboveground biomass stocks that were even comparable with tropical forests. There were not enough observations for boreal deciduous broadleaf forest, but given their presence in largely early successional stages in most boreal regions, we believe that the validation for boreal coniferous forest is adequate for our purpose.

For the time periods around 2010, the spatial distribution of simulated forest aboveground biomass largely agreed with those given by satellite-based observations (four data sets; Supplementary Figure 6). Linear regressions accounting for all grid cells across the globe indicated that the model simulation agreed with three of the four observations with an error smaller than 10%. Pixel-scale comparisons showed that for most grid cells the error between the model and observations was within 20%, with mismatches largely dominated by model overestimation (Supplementary Figure 6). The overestimation occurred mainly in tropical forests, temperate forests in eastern China and eastern North America, and boreal forests in western and central Eurasia. The overestimation of biomass in tropical forest was consistent with the overestimation of forest growth as shown in Supplementary Figure 7b. The model also showed underestimation in the larch forest belt over eastern Siberia and temperate forests in Southeast Australia.

However, it should be noted that the model errors presented here contain all errors and uncertainties including those in model structure and parameterization and all forcing data being used, and in particular, the uncertainties in the LUC forcing data. For example, accounting for shifting cultivation in the SC-sensitivity run further reduced the tropical forest biomass and relieved the issue of overestimation. The tendency for ORCHIDEE to overestimate forest biomass might be linked to its incapability to represent late-successional forest species change and various mortality agents including gap formation, selective logging and disturbances from wind, insect, and diseases. This limitation is, however, shared by most DGVMs and is an active area of model development. We expect that more forest loss allocated to high-biomass areas and the overestimation of tropical forest biomass would yield a high bias in E_{fire} and E_{legacy} fluxes.

Supplementary Note 4 Matching the global composition of primary and secondary forest for 2015 with that derived by the HN2017 bookkeeping model for the baseline simulation

Supplementary Figure 7 shows the forest area dynamics of different age classes as simulated by the ORCHIDEE baseline simulation between 1700 and 2015. As of 1700 after the initial model spin-up and prior to the start of the transient simulation, all forests were found in the oldest forest age class (Age 6). Forests other than the oldest age class started to emerge from 1701 as a result of agricultural abandonment and forest regrowth after wood harvest and as a result of afforestation/reforestation. Their area continued to grow with time as human beings perturbed more and more ‘intact forests’ of Age 6. Historical deforestation accounted for a net forest loss of 9.9 million km^2 over 1700–2015.

We grouped the two oldest age classes, Age 6 and Age 5, whose wood mass exceeded 90% of the attainable maximum wood mass determined under the pre-industrial conditions, as ‘intact forest’ and the remaining four younger age classes as ‘secondary forest’. The secondary forest area has continued to grow since 1701 and reached a peak in 1953, and then declined as part of the Age 4 forests were re-classified as Age 5 because their wood mass exceeded the corresponding age class

threshold. As of 2015, our modeled secondary forests accounted for 17% of the global total forest area, slightly lower than the 19% obtained by the HN2017 bookkeeping model[5]. To ensure the consistency in forest composition with the HN2017 study, we manually regrouped part of the 'Age 5' forests into secondary forests by linearly increasing the secondary forests percentage from 1953 and making it reach the 19% target in 2015.

Supplementary Note 5 Comparing estimated secondary forest carbon sink with Pugh et al. 2019 for the baseline simulation

Our simulated carbon sink in the global secondary forest, when emissions from the wood product degradation were not accounted for, was 0.83 Pg C yr^{-1} for 1990–2015, or 0.53 Pg C yr^{-1} after accounting for wood product degradation. These values were for a given secondary-forest percentage of 19% of the total forest area, which was set to be consistent with HN2017. A recent study by Pugh et al.[6] reported a global secondary forest sink of 1.30 (1.03–1.96) Pg C yr^{-1} by assuming all forests with an age less than 140 years as secondary forest, which accounts for 61.4% of the total forest area. Adjusting the secondary forest to have the same proportion as Pugh et al.[6] and accounting for the total forest area difference between Pugh et al. [6] and ORCHIDEE (42.8 Mkm^2 vs. 48.7 Mkm^2) would yield a regrowth sink of 1.69 Pg C yr^{-1} in our study, well agreeing with the value reported by Pugh et al.[6].

Supplementary Note 6 Scale Issues related to the areas undergoing land use change

We included three major forms of land use change in this study: large-scale net land use change including deforestation or afforestation/reforestation, wood harvest at a large scale, and local-scale shifting cultivation shown as simultaneous land transitions of equal areas between two given land use types over the same grid cell. As the classification of land use type and quantification of their transitions are intrinsically scale-dependent, the areas subjected to LUC activities are expected to be also scale-dependent. This should be reflected in the spatial resolution of the LUC-forcing datasets used in our simulation, be it explicitly stated or not.

In ORCHIDEE, both directions of land flow between two given land use types were explicitly represented. The uncertainty of areas subjected to LUC was thus independent of the spatial resolution of the model simulation, because land transitions in the original LUC forcing data, regardless of their original spatial resolution, could always be summed and re-implemented over the model grids (which was what we actually did for this study). Therefore in the following we will ignore the model resolution and focus on the influence of spatial resolution on LUC areas in each original forcing dataset used.

The areas of net land use change and forest wood harvest in our simulation should fairly reflect the large-scale change only. As is explained in Section 1, changes in agricultural area in our LUC-forcing data were derived from the LUH2 dataset with a 0.25-degree spatial resolution, whose agricultural areas were further derived from the HYDE3.2 dataset. Although the HYDE3.2 dataset has a spatial resolution of 5', it was built using an allocation algorithm by disintegrating estimates of agricultural land area at national or subnational scale[3]. The latter were either derived from FAO national statistical data or based on calculations of national population and per capita land use. Likewise, the changes of forest area and annual volumes of wood harvest were also derived from FAO statistical data for recent periods or reconstructed from historical narratives and national statistics.

Shifting cultivation represents local-scale subsistence agricultural practice. It was included in both the LUH1 and LUH2 datasets. In the LUH1 dataset, shifting cultivation was derived by a specific land use modeling tool that addressed the complex process of land use allocation, with assumptions on the fallow length of tropical shifting cultivation[11]. A similar approach has been used in the LUH2 data[1]. Therefore, we assumed that shifting cultivation extracted from LUH2 data represented mainly local-scale land use change activities.

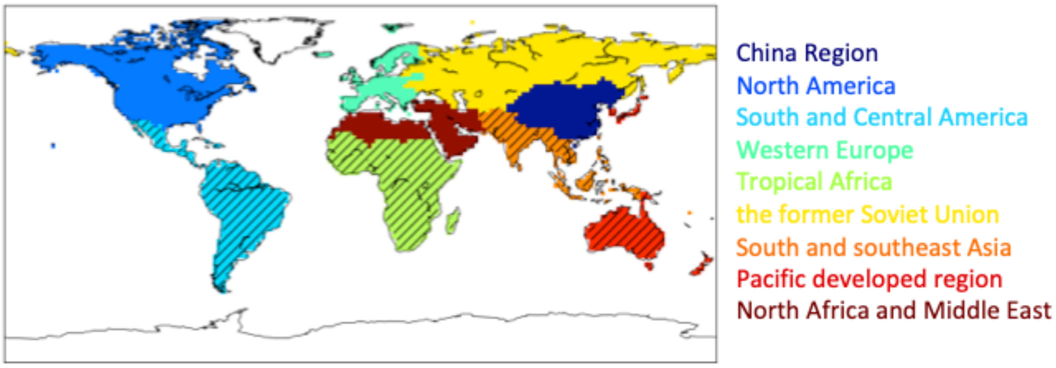

Supplementary Figure 1 The nine regions for which the ORCHIDEE input data of historical forest area changes and wood harvest biomass are constrained by those used in HN2017. Hatched regions indicate regions that are dominated by deforestation loss during the 2000-2014 (used in Supplementary Figure 5).

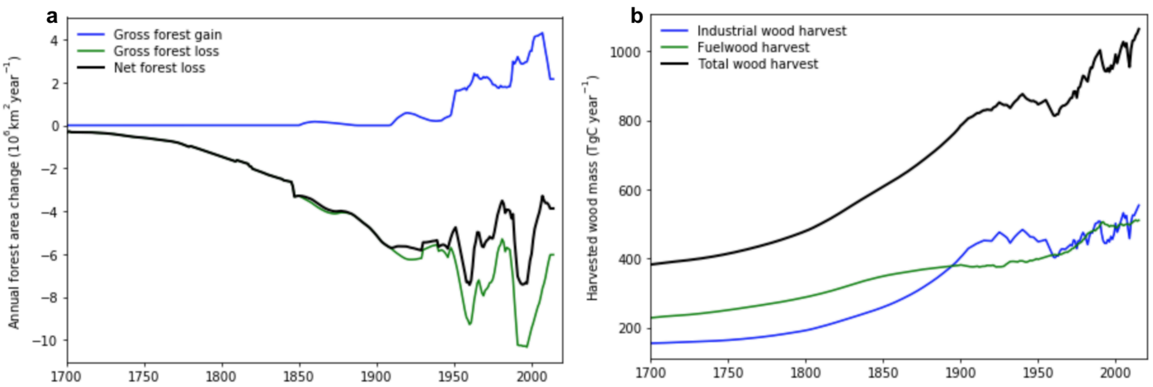

Supplementary Figure 2 Forcing data for forest area change and wood harvest used in ORCHIDEE. **(a)** global annual forest area change, and (**b**) harvested wood mass for industrial purposes and fuel wood. The data are the same as used in the bookkeeping model in HN2017.

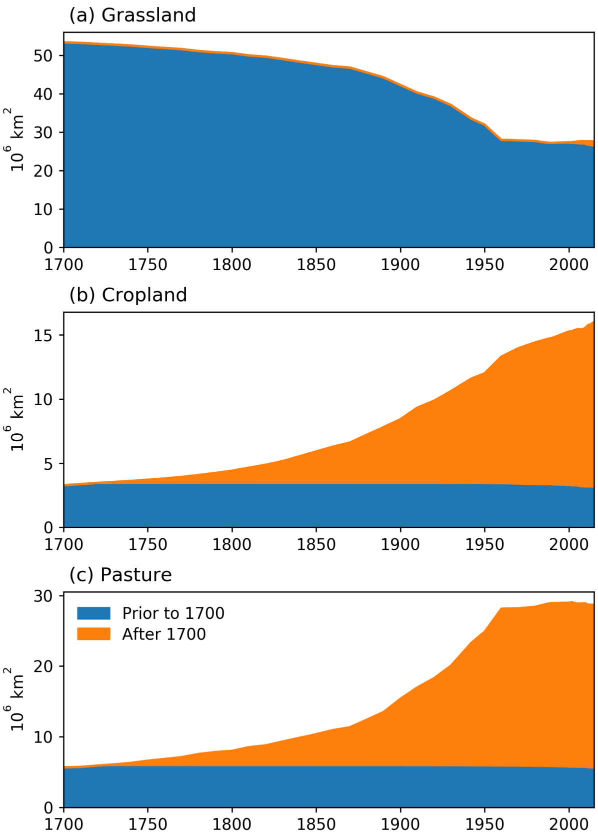

Supplementary Figure 3 Temporal changes in the area of herbaceous vegetation at various management stages from the baseline simulation. All areas are shown as stacked area plots. (a) Changes in the areas of intact (pre-1700) and recovering natural grassland (post-1700) as a result of agricultural abandonment. (b) Changes in the areas of post-industrial cropland (post-1700) versus permanent cropland (pre-1700). (c) Changes in the areas of post-industrial pasture (post-1700) versus permanent pasture (pre-1700). Intact grassland area showed continual decrease since 1700 as a result of its conversion to agricultural land (cropland and pasture), while the area of recovering grassland remained low and little changed, indicating low quantity of agricultural abandonment. Permanent cropland and pasture remained almost constant throughout the whole simulation, demonstrating that they were well separated from cropland and pasture established after 1700, both of which showed continuous increase since then. This figure demonstrates the clear separation of land cohorts at different management stages at a sub-grid scale in the improved ORCHIDEE model.

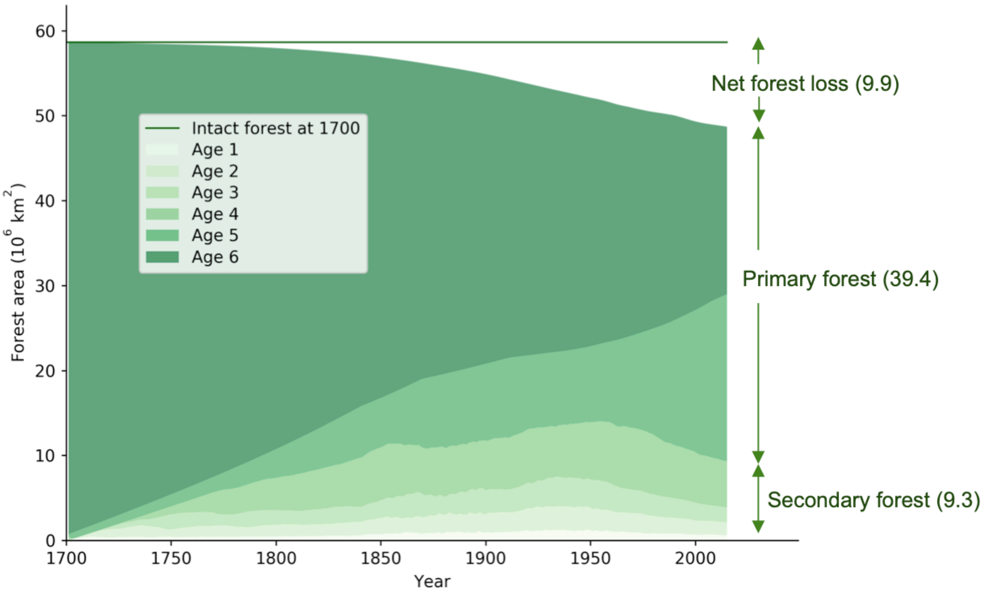

Supplementary Figure 4 Modeled forest areas of different age classes from the baseline simulation. Different forest age classes were regrouped into primary or intact forest and secondary forest. The fraction of intact forest was adjusted to be the same as HN2017.

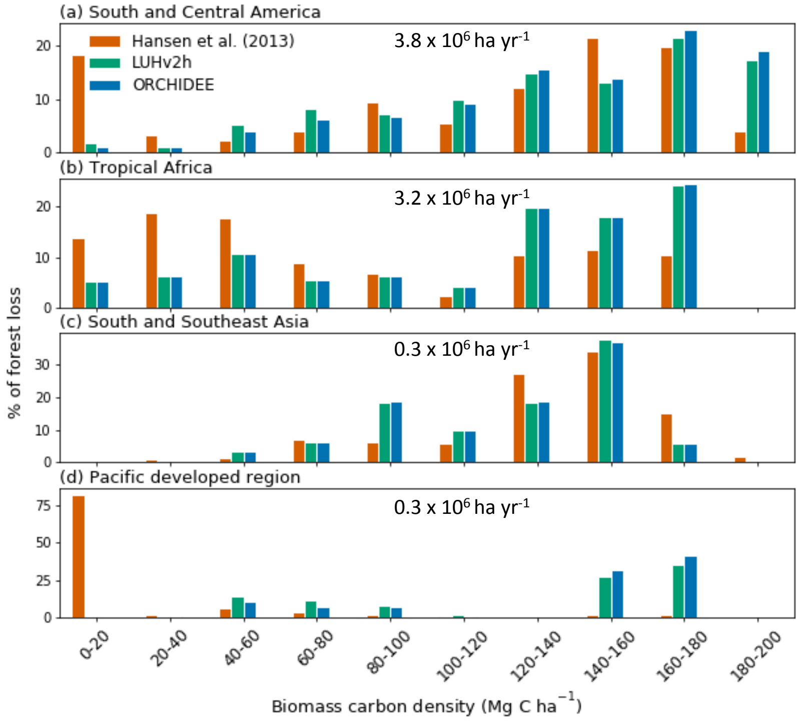

Supplementary Figure 5 Forest loss distribution along the modeled gradients of above biomass density. Orange bars, as obtained from satellite-derived gross tree cover loss (ref[4]); blue bars, as derived from the spatial distribution of forest loss in the LUH2 data set; and green bars, as implemented in ORCHIDEE which was derived by integrating the HN2017 regional forest area change and the spatial distribution of forest loss in LUH2. The data are shown for 2000-2014. Panels of (**a**) to (**d**) show data for the four regions over which deforestation loss dominates (hatched area in Supplementary Figure 1). The number on each sub-panel shows the mean annual area of net forest loss implemented in ORCHIDEE.

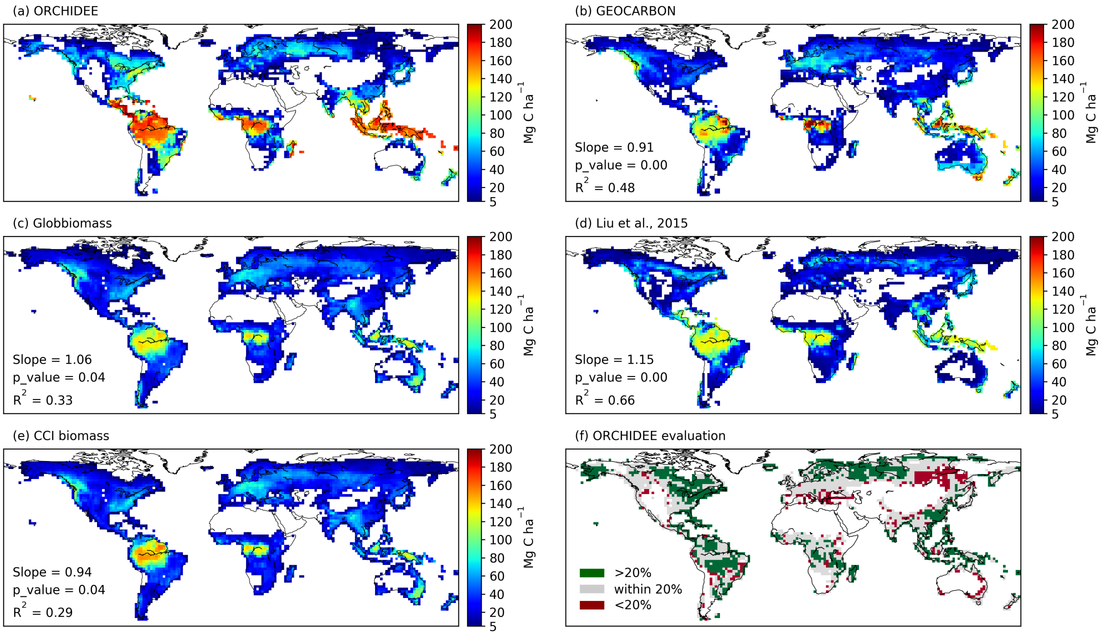

Supplementary Figure 6 Evaluation of ORCHIDEE-simulated aboveground biomass carbon density with multiple satellite-based observations. The model result is from the baseline simulation. (**a**) ORCHIDEE model result for the year of 2010. **(b)** GEOCARBON (ref[12]) for the decade of 2000-2009. **(c)** Globbiomass for 2010 (https://doi.pangaea.de/10.1594/PANGAEA.894711). **(d)** Liu et al. 2015 for the year 2000 (ref[13]). **(e)** CCI biomass for the year of 2017 (http://cci.esa.int/biomass). **(f)** Evaluation of ORCHIDEE in light of the observation data sets. We assumed a 20% uncertainty for satellite-based observation data. Green (red) color means that the aboveground biomass carbon density simulated by ORCHIDEE is 20% higher (lower) than the highest (lowest) value of the satellite-based observations. All gray grid cells were considered to have acceptable simulated aboveground biomass carbon.

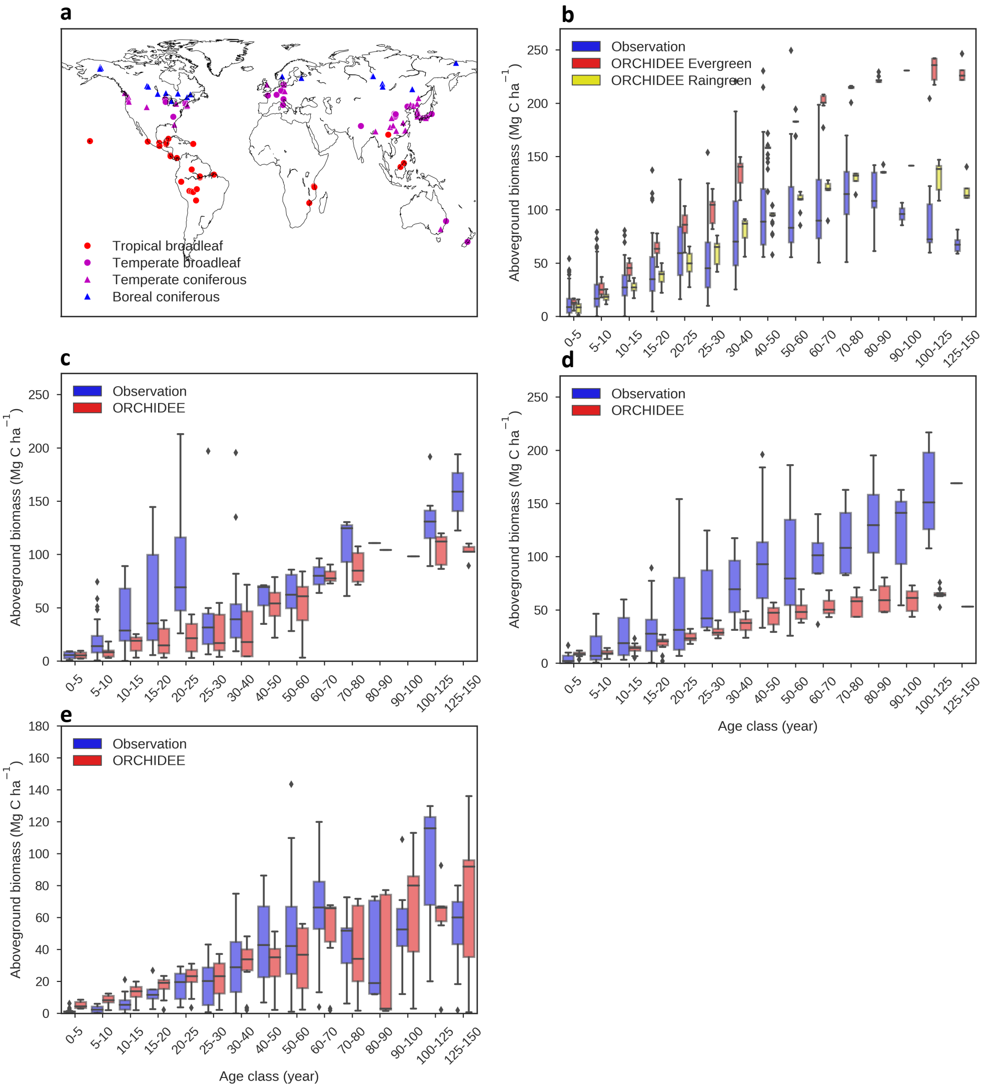

Supplementary Figure 7 Comparison of modeled and observed forest aboveground biomass-age relationships. (**a**) Spatial distribution of sites where chronosequence-based observations were compiled in this study (see references in Supplementary Table 2). Comparisons are shown for tropical broadleaf forest (n=513) (**b**), temperate broadleaf forest (n=144) (**c**), temperate coniferous forest (n=209) (**d**), and boreal coniferous forest (n=225) (**e**)**.** For tropical broadleaf forest, simulated forest biomass growth by both evergreen and raingreen forest PFTs in ORCHIDEE were provided. Model values were extracted from the grid cells where observation sites were located with corresponding forest ages. The center line of the boxplot shows the median value, with box limits showing upper land lower quantiles and whiskers showing 1.5x interquantile range and points showing outliers.

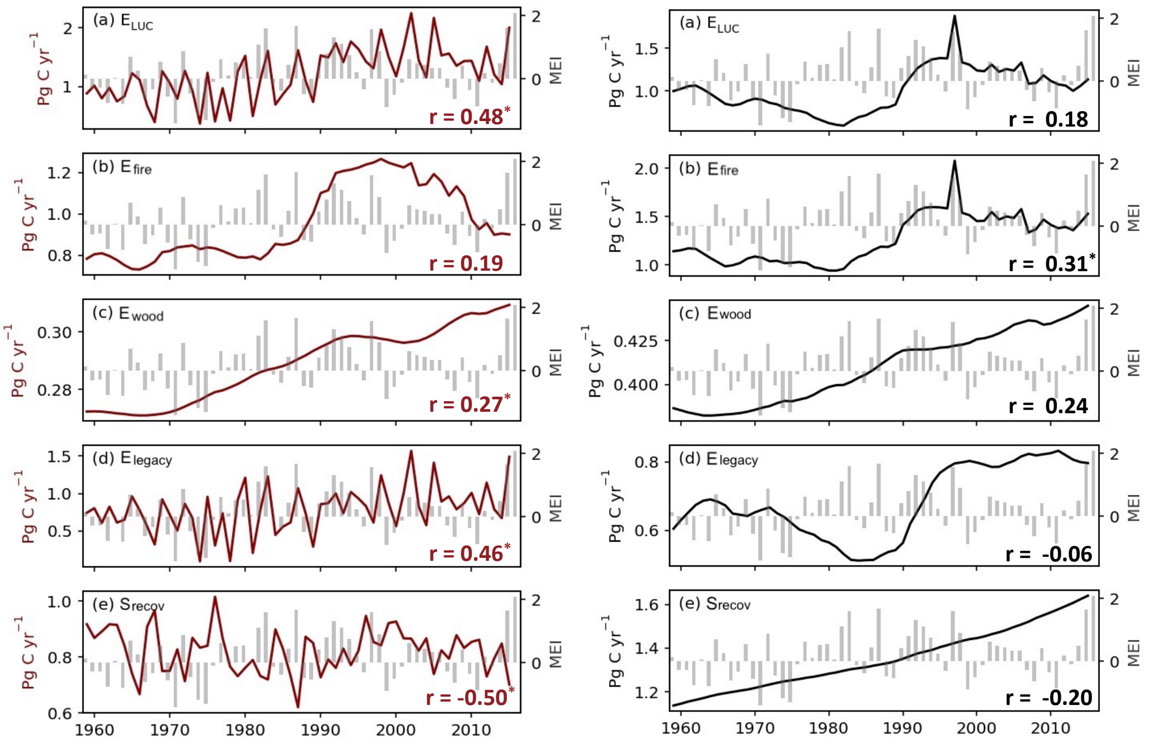

Supplementary Figure 8 E_{LUC} and its component fluxes (lines) and their relationships to the ENSO climate variation (bars). Model results were from the baseline simulation. Left panels show ORCHIDEE results and right panels show HN2017 results. Their Pearson correlation coefficients (r) with the Multivariate ENSO Index (MEI) are also shown, with the asterisk indicating a p-value less than 0.05. Positive MEI values indicate the hot and dry El Niño phase and the reverse indicate the cool and wet La Niña phase. See main text Fig. 1 for explanation of component fluxes of E_{LUC} shown in (b)–(e).

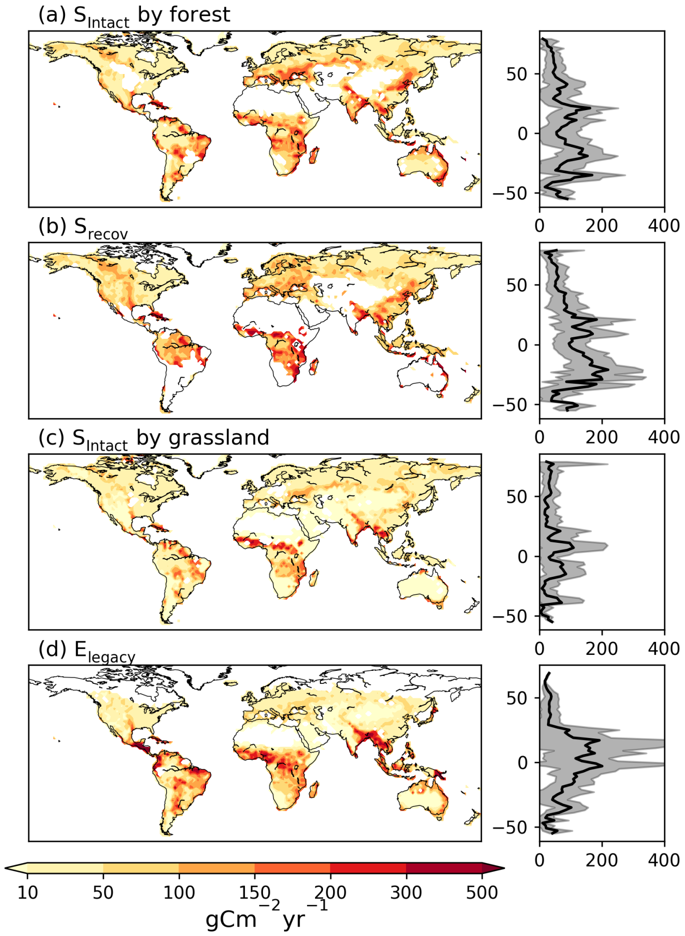

Supplementary Figure 9 Standard deviation of annual land-atmosphere carbon fluxes over lands of different management status for 1959–2015 from the baseline simulation. a, Intact land sink contributed by intact forest. b, Carbon sink over recovering secondary forest and grassland. c, Intact land sink contributed by intact grassland. d, Legacy carbon emissions over recently established agricultural land. Solid dark lines indicate mean value for all pixels of each 2-degree latitude band and shaded areas indicate the standard deviation among these pixels.

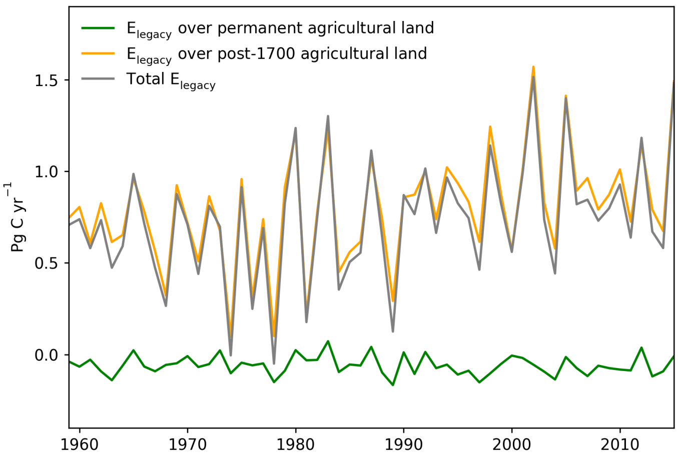

Supplementary Figure 10 E_{legacy} as carbon emissions (gray line) over the post-1700 agricultural land (orange line) that was partly offset by a small carbon sink with low IAV over the permanent pre-1700 agricultural land (green line). The mean value of E_{legacy} over 1959-2015 was 0.73+/-0.33 Pg C yr^{-1} (uncertainty being the standard deviation). The mean value of carbon source (i.e., emissions) over post-1700 agricultural land was 0.79+/-0.31 Pg C yr^{-1}. The mean value of carbon sink over permanent pre-1700 agricultural land was 0.06+/-0.05 Pg C yr^{-1}. Including the carbon sink over permanent agricultural land into E_{legacy} had only a small influence in its magnitude, allowing the E_{legacy} simulated by ORCHIDEE to still be able to be compared with the HN2017 estimate. The carbon balance over permanent agricultural land was not taken into account in the latter.

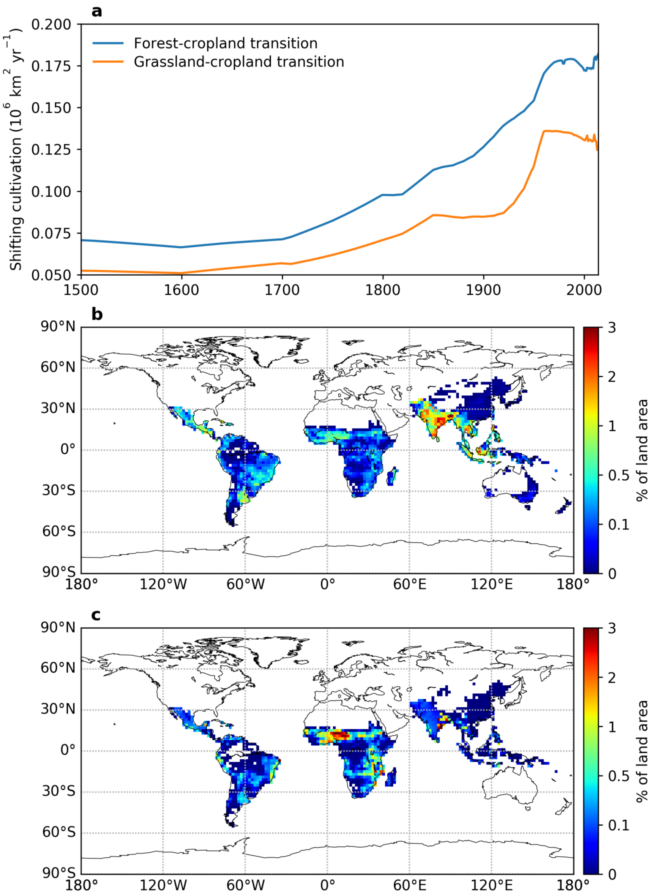

Supplementary Figure 11 Spatial distribution and historical changes of areas subject to shifting cultivation. Shifting cultivation was extracted from the LUH2 dataset as simultaneous land transitions with equal areas between forest (or natural grassland) and cropland. (**a**) Historical changes of areas subject to shifting cultivation in the LUH2 dataset. (**b**) Spatial distribution of areas subjected to shifting cultivation between forest and cropland averaged annually for 1500-2015. (**c**) Spatial distribution of land transitions between natural grassland and cropland averaged for 1500-2015. No transitions between forest (or natural grassland) and pasture for shifting cultivation were included in LUH2.

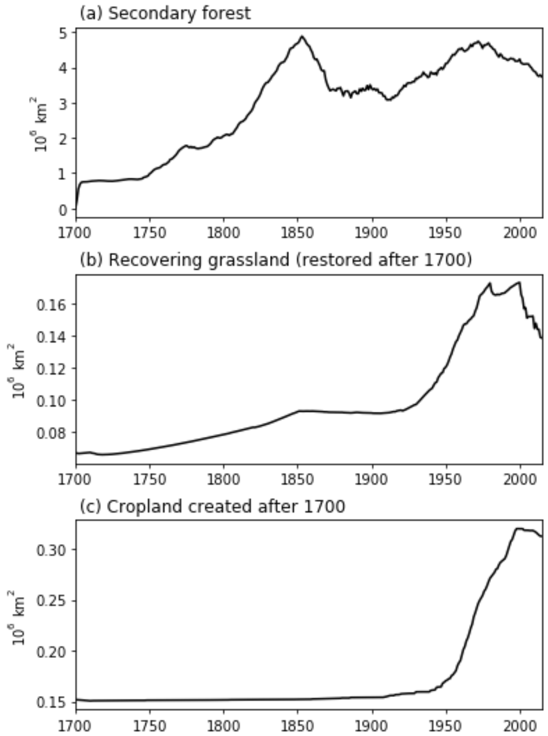

Supplementary Figure 12 Difference in areas of secondary forest (a), secondary grassland (b), and post-industrial cropland that were created after 1700 (c) between the SC-sensitivity run and the baseline run. Positive values indicate that including shifting cultivation yielded higher areas in these land use types. As the total areas of forest, grassland and cropland were the same between the two simulations, higher areas in secondary lands implicate lower areas in 'intact' land, i.e., intact forest, grassland, and permanent agricultural land existing prior to 1700.

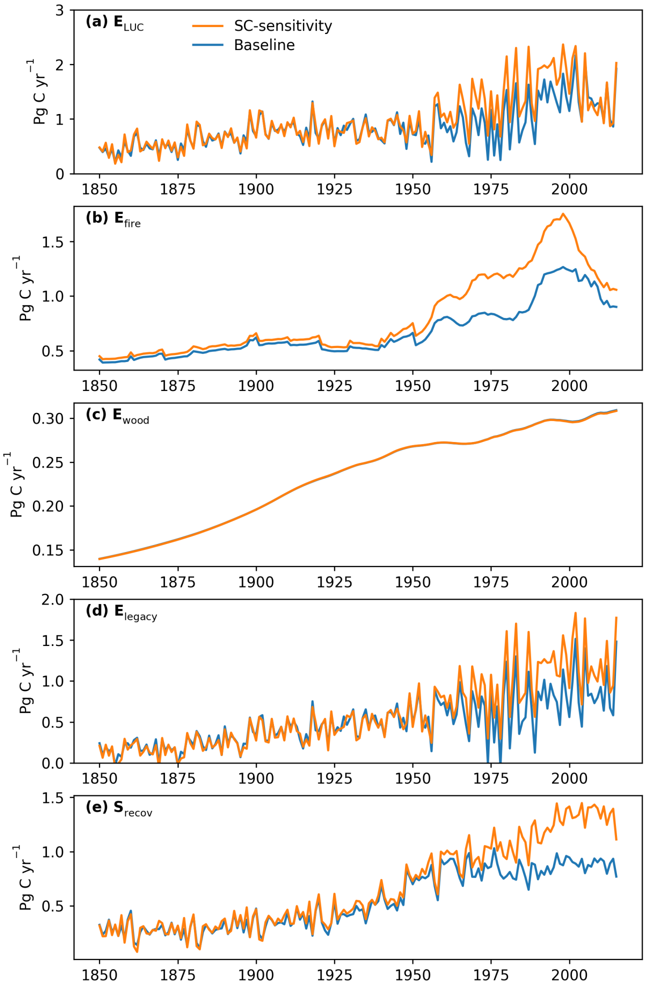

Supplementary Figure 13 Fluxes of E_{LUC}, E_{fire}, E_{wood}, E_{legacy}, and S_{recov} as simulated by the baseline and SC-sensitivity simulations by the ORCHIDEE model over the period of 1850-2015.

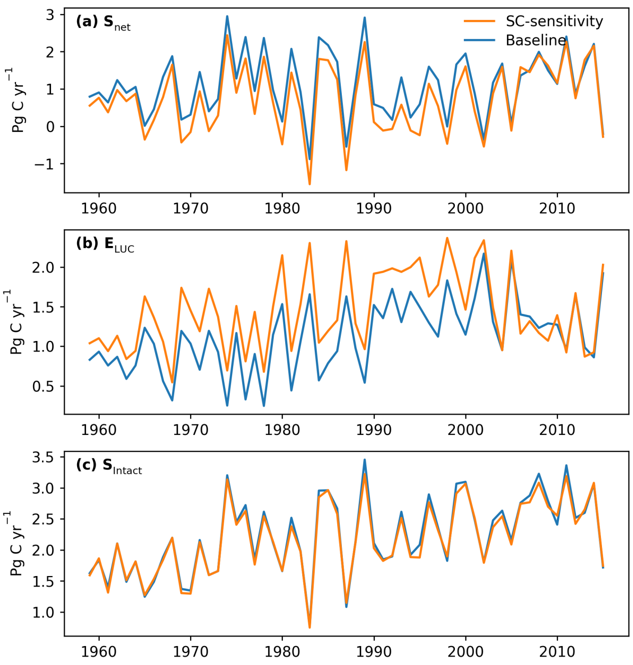

Supplementary Figure 14 Carbon fluxes of E_{LUC}, S_{net}, and S_{Intact} as simulated by the baseline and SC-sensitivity simulations by the ORCHIDEE model for 1959-2015.

Supplementary Table 1 Detailed descriptions for E_{LUC} flux components, their associated processes with land use change, and parameterizations in HN2017 study and ORCHIDEE simulation.

Flux	HN2017	ORCHIDEE	Remark
E_{fire}	• Aboveground biomass burning when forest is cleared for agricultural land<br>• Fuel wood harvest<br>• The first-year releases from slash and soil carbon when forest is converted to agricultural land	• Aboveground biomass burning when forest is cleared for agricultural land<br>• Fuel wood harvest	• Both assume 50% of aboveground biomass is burned when forest is converted to cropland, and 67% burned when forest is converted to pasture.<br>• Both assume 100% of aboveground woody biomass is burned for fuel wood harvest.
E_{wood}	• Industrial wood product decay	• Industrial wood product decay	• Both assume that for tropical forest, 10% of aboveground woody mass is used as wood product with a 10-year residence time, and 53% is used as wood product with a 100-year residence time. For temperate and boreal forest, the respective fractions are 19% and 47%.
E_{legacy}	• The decomposition of slash and soil carbon as a result of forest clearing for agricultural land<br>• The decomposition of slash resulting from the unused aboveground woody mass in industrial wood harvest, which is 37% for tropical forest, and 34% for temperate and boreal forest. Wood harvest does not impact soil carbon dynamics.	• Net carbon flux over agricultural land, which is assumed to be dominated by that of legacy slash and soil carbon release as a result of forest clearing for agricultural land	• The first-year releases from slash and soil carbon when forest is converted to agricultural land, which is included in E_{fire} in HN2017, is included in E_{legacy} in ORCHIDEE. But the influence is expected to be small.

S_{recov}	• Biomass and soil carbon accumulation in secondary forest recovering from agricultural abandonment.<br>• Biomass carbon accumulation in secondary forest recovering from wood harvest. There are no changes in soil carbon.	• Net carbon flux over secondary forest recovering from agricultural abandonment<br>• Net carbon flux over secondary forest recovering from wood harvest	• The S_{recov} by ORCHIDEE also includes the effect of slash decomposition resulting from the unused aboveground woody mass in industrial wood harvest, whose fractions are the same as in HN2017.

Supplementary Table 2 References for the chronosequence-based forest growth database compiled in this study. The table could be found at the end of the current file.

Lead Author	Year	Journal name	Article title	Forest type
Uhl	1987	The Journal of Ecology	Factors controlling succession following slash-and-burn agriculture in Amazonia	Tropical broadleaf
Saldarriaga	1988	The Journal of Ecology	Long-term chronosequence of forest succession in the upper Rio Negro of Colombia and Venezuela	Tropical broadleaf
Szott	1994	Forest Ecology and Managment	Biomass and litter accumulation under managed and natural tropical fallows	Tropical broadleaf
Alves	1997	Global Change Biology	Biomass of primary and secondary vegetation in Rondonia, Western Brazilian Amazon	Tropical broadleaf
Hughes	1999	Ecology	Biomass, carbon, and nutrient dynamics of secondary forests in a humid tropical region of Mexico	Tropical broadleaf
Aide	2000	Restoration Ecology	Forest regeneration in a chronosequence of tropical abandoned pastures Implications for restoration ecology	Tropical broadleaf
Steininger	2000	Tropical Ecology	Secondary forest structure and biomass following short and extended land-use in central and southern Amazonia	Tropical broadleaf
Lucas	2002	International Journal of Remote Sensing	Forest regeneration on abandoned clearances in central Amazonia	Tropical broadleaf
vieira	2003	Remote Sensing of Environment	Classifying successional forests using Landsat spectral properties and ecological characteristics in eastern Amazoˆnia	Tropical broadleaf
Read	2003	Ecological Applications	Recovery of biomass following shifting cultivation in dry tropical forests of the Yucatan	Tropical broadleaf
Pena-Claro	2003	Biotropica	Changes in Forest Structure and Species Composition during Secondary Forest Succession in the Bolivian Amazon	Tropical broadleaf
Ryan	2004	Ecological Monographs	An experimental test of the causes of forest growth decline with stand age	Tropical broadleaf
Jepsen	2006	Forest Ecology and Management	Above-ground carbon stocks in tropical fallows, Sarawak, Malaysia	Tropical broadleaf

Marin-Spiotta	2007	Ecological Applications	Long-term patterns in tropical reforestation: plant community composition and aboveground biomass accumulation	Tropical broadleaf
Vargas	2008	Global Change Biology	Biomass and carbon accumulation in a fire chronosequence of a seasonally dry tropical forest	Tropical broadleaf
Lebrija-Trejos	2008	Biotropica	Successional change and resilience of a very dry tropical deciduous forest following shifting agriculture	Tropical broadleaf
Williams	2008	Forest Ecology and Management	Carbon sequestration and biodiversity of re-growing miombo woodlands in Mozambique	Tropical broadleaf
Powers	2009	Forest Ecology and Managment	Diversity and structure of regenerating tropical dry forests in Costa Rica: Geographic patterns and environmental drivers	Tropical broadleaf
Becknell	2014	Canadian Journal of Forest Research	Stand age and soils as drivers of plant functional traits and aboveground biomass in secondary tropical dry forest	Tropical broadleaf
Broadbent	2014	Plos one	Integrating Stand and Soil Properties to Understand Foliar Nutrient Dynamics during Forest Succession Following Slash-and-Burn Agriculture in the Bolivian Amazon	Tropical broadleaf
Mora	2014	Biotropica	Testing chronosequences through dynamic approaches: time and site efects on tropical dry forest succession	Tropical broadleaf
Aryal	2014	Agriculture, Ecosystems and Environment	Carbon stocks and changes in tropical secondary forests of southern Mexico	Tropical broadleaf
McNicol	2015	Ecological Applications	How resilient are African woodlands to disturbance from shifting cultivation?	Tropical broadleaf
Du	2015	Forests	Carbon Storage in a Eucalyptus Plantation Chronosequence in Southern China	Tropical broadleaf
Fujiki	2016	Journal of Tropical Ecology	Plant communities and ecosystem processes in a succession-altitude matrix after shifting cultivation in the tropical montane forest zone of northern Borneo	Tropical broadleaf
Buzzard	2016	Functional Ecology	Re-growing a tropical dry forest: functional plant trait composition and community assembly during succession	Tropical broadleaf

Jones	2019	Science of the Total Environment	Above- and belowground carbon stocks are decoupled in secondary tropical forests and are positively related to forest age and soil nutrients respectively	Tropical broadleaf
Forrest	1970	Journal of Applied Ecology	Organic matter changes in an age series of Pinus radiata plantations	Temperate coniferous
Long	1975	Journal of Applied Ecology	Aboveground biomass of understorey and overstorey in an age sequence of 4 Douglas-Fir stands	Temperate coniferous
Gholz	1982	Ecology	Organic matter production and distribution in slash pine plantations	Temperate coniferous
Das	1987	Forest Ecology and Management	Above-ground biomass and nutrient contents in an age series of Khasi Pine	Temperate coniferous
Chen	1998	Forest Ecology and Management	Biomass and nutrient distribution in a Chinese-fir plantation chronosequence in Southwest Hunan, China	Temperate coniferous
Janisch	2002	Tree Physiology	Successional changes in live and dead wood carbon stores: implications for net ecosystem productivity	Temperate coniferous
Law	2003	Global Change Biology	Changes in carbon storage and fluxes in a chronosequence of ponderosa pine	Temperate coniferous
Hooker	2003	Ecological Applications	Forest ecosystem carbon and nitrogen accumulation during the first century after agricultural abandonment	Temperate coniferous
Shutou	2004	Ecological Research	Change in soil carbon cycling for stand development of Japanese cedar (Cryptomeria japonica) plantations following clear-cutting	Temperate coniferous
Zerva	2005	Forest Ecology and Management	Soil carbon dynamics in a Sitka spruce (Picea sitchensis (Bong.) Carr.) chronosequence	Temperate coniferous
Wang	2006	Journal of Integrative Plant Biology	Soil carbon changes following afforestation with Olga Bay larch (Larix olgensis Henry) in northeastern China	Temperate coniferous
Humphreys	2006	Agricultural and Forest Meteorology	Carbon dioxide fluxes in coastal Douglas-fir stands at different stages of development after clearcut harvesting	Temperate coniferous
Peichl	2007	Biogeochemistry	Concentrations and fluxes of dissolved organic carbon in an age-sequence of white pine forests in Southern Ontario, Canada	Temperate coniferous

Compton	2007	Ecosystems	Ecosystem N distribution and δ 15 N during a century of forest regrowth after agricultural abandonment	Temperate coniferous
Tateno	2009	Journal of Forestry Research	Biomass allocation and nitrogen limitation in a Cryptomeria japonica plantation chronosequence	Temperate coniferous
Peichl	2010	Agricultural and Forest Meteorology	Biometric and eddy-covariance based estimates of carbon fluxes in an age-sequence of temperate pine forests	Temperate coniferous
Jin	2010	Science China(Life Sciences)	Carbon and nitrogen storage in an age-sequence of Pinus densiflora stands in Korea	Temperate coniferous
Zhao	2011	Journal of Forestry Research	Aboveground biomass and nutrient allocation in an age-sequence of Larix olgensis plantations	Temperate coniferous
Li	2011	Journal of Plant Biology	Biomass and Carbon Storage in an Age-Sequence of Korean Pine (Pinus koraiensis) Plantation Forests in Central Korea	Temperate coniferous
Chen	2012	Forest Ecology and Management	Carbon storage in a chronosequence of Chinese fir plantations in southern China	Temperate coniferous
Li	2013	Journal of Forest Research	Biomass and carbon storage in an age-sequence of Japanese red pine (Pinus densiflora) forests in central Korea	Temperate coniferous
Kim	2013	Journal of Korean Society of Forest Science	Allometric Equations and Biomass Expansion Factors in an Age-sequence of Black Pine (Pinus thunbergii) Stands	Temperate coniferous
Ma	2013	Journal of Forestry Research	Carbon stock in Korean larch plantations along a chronosequence in the Lesser Khingan Mountains, China	Temperate coniferous
Li	2014	The Forestry Chronicle	Patterns of biomass allocation in an age-sequence of secondary Pinus bungeana forests in China	Temperate coniferous
Justine	2015	Forests	Biomass Stock and Carbon Sequestration in a Chronosequence of Pinus massoniana Plantations in the Upper Reaches of the Yangtze River	Temperate coniferous
Zhou	2015	Journal of Forestry Research	Biomass production, nutrient cycling and distribution in age-sequence Chinese fir (Cunninghamia lanceolate) plantations in subtropical China	Temperate coniferous
Xie	2016	Dendrobiology	Biomass partition and carbon storage of Cunninghamia lanceolata chronosequence plantations in Dabie Mountains in East China	Temperate coniferous

Urbano	2017	Forest Ecology and Management	Carbon dynamics and structural development in recovering secondary forests of the northeastern U.S	Temperate coniferous
Zeng	2018	Forests	Distribution Changes of Phosphorus in Soil-Plant Systems of Larch Plantations across the Chronosequence	Temperate coniferous
Boring	1984	Journal of Ecology	The Role of Black Locust (Robinia Pseudo-Acacia) in Forest Succession	Temperate broadleaf
Ruark	1987	Canadian Journal of Forest Research	Biomass, net primary production, and nutrient distribution for an age sequence of Populustremuloides ecosystems	Temperate broadleaf
Kawaguchi	1989	Ecological Research	Carbon-cycling changes during regeneration of a decinduous broadleaf forest after clear-cutting II. Aboveground net production	Temperate broadleaf
Lodhiyal	1995	Annals of Botany	Structure and function of an age series of poplar plantations in Central Himalaya	Temperate broadleaf
Smith	1999	Forest Science	Age-related changes in production and below-ground carbon allocation in Pinus contorta forests	Temperate broadleaf
Clinton	2002	Canadian Journal of Forest Research	Nitrogen storage and availability during stand development in a New Zealand Nothofagus forest	Temperate broadleaf
Vesterdal	2002	Forest Ecology and Management	Change in soil organic carbon following afforestaton of former arable land	Temperate broadleaf
Claus	2005	Canadian Journal of Forest Research	Effect of stand age on fine-root biomass and biomass distribution in three European forest chronosequences	Temperate broadleaf
Tedeschi	2006	Global Change Biology	Soil respiration in a Mediterranean oak forest at different developmental stages after coppicing	Temperate broadleaf
Alberti	2008	Forestry	Forest ecosystem carbon accumulation during a secondary succession in the Eastern Prealps of Italy	Temperate broadleaf
Turner	2008	Forest Ecology and Management	Nutrient cycling in age sequences of two Eucalyptus plantation species	Temperate broadleaf
Tang	2009	Global Change Biology	Soil carbon fluxes and stocks in a Great Lakes forest chronosequence	Temperate broadleaf
Mao	2010	Plant Soil	Soil organic carbon and nitrogen stocks in an age-sequence of poplar stands planted on marginal agricultural land in Northeast China	Temperate broadleaf

Genet	2010	Tree Physiology	Age-related variation in carbon allocation at tree and stand scales in beech (Fagus sylvatica L.) and sessile oak (Quercus petraea (Matt.) Liebl.) using a chronosequence approach	Temperate broadleaf
Simon	2012	European Journal of Forest Research	Carbon stocks and net ecosystem production changes with time in two Italian forest chronosequences	Temperate broadleaf
Lu	2013	Biogeosciences	Soil organic carbon dynamics of black locust plantations in the middle Loess Plateau area of China	Temperate broadleaf
Li	2015	Plos one	Spatial Variation in the Storages and Age Related Dynamics of Forest Carbon Sequestration in Different Climate Zones—Evidence from Black Locust Plantations on the Loess Plateau of China	Temperate broadleaf
Hyodo	2016	Journal of Forest Research	Changes in aboveground and belowground properties during secondary natural succession of a cool-temperate forest in Japan	Temperate broadleaf
Zhang	2017	Journal of Forestry Research	Ecosystem carbon and nitrogen storage following farmland afforestation with black locust (Robinia pseudoacacia) on the Loess Plateau,China	Temperate broadleaf
Schulze	1999	Global Change Biology	Productivity of forests in the Eurosiberian boreal region and their potential to act as a carbon sink - a synthesis	Boreal coniferous
Rothstein	2004	Canadian Journal of Forest Research	Loss and recovery of ecosystem carbon pools following stand-replacing wildfire in Michigan jack pine forests	Boreal coniferous
Howard	2004	Global Change Biology	Effects of logging on carbon dynamics of a jack pine forest in Saskatchewan, Canada	Boreal coniferous
Martin	2005	Global Change Biology	Carbon pools in a boreal mixedwood logging chronosequence	Boreal coniferous
Clarke	2007	Plant and Soil	Dissolved organic carbon concentrations in four Norway spruce stands of different ages.	Boreal coniferous
Simard	2007	Ecological Applications	Forest productivity decline caused by successional paludification of boreal soils	Boreal coniferous
Borja	2008	Tree Physiology	Stand age and fine root biomass, distribution and morphology in a Norway spruce chronosequence in southeast Norway	Boreal coniferous

Mack	2008	Ecosystems	Recovery of Aboveground Plant Biomass and Productivity After Fire in Mesic and Dry Black Spruce Forests of Interior Alaska	Boreal coniferous
mkhabela	2009	Agricultural and Forest Meteorology	Comparison of carbon dynamics and water use efficiency following fire and harvesting in Canadian boreal forests	Boreal coniferous
Karu	2009	Canadian Journal of Forest Research	Carbon sequestration in a chronosequence of Scots pine stands in a reclaimed opencast oil shale mine	Boreal coniferous
Goulden	2011	Global Change Biology	Patterns of NPP, GPP, respiration, and NEP during boreal forest succession	Boreal coniferous
Alexander	2012	Ecosystems	Carbon Accumulation Patterns During Post-Fire Succession in Cajander Larch (Larix cajanderi) Forests of Siberia	Boreal coniferous
Park	2014	Forest Ecology and Management	Carbon storage and stand conversion in a pine-dominated boreal forest landscape	Boreal coniferous
Taylor	2014	Ecosystems	Decline in Net Ecosystem Productivity Following Canopy Transition to Late-Succession Forests	Boreal coniferous
Baret	2015	Forest Ecology and Management	Long-term changes in belowground and aboveground resource allocation of boreal forest stands	Boreal coniferous
Gao	2017	Ecosystems	Carbon Storage Declines in Old Boreal Forests Irrespective of Succession Pathway	Boreal coniferous
Senez-Gagnon	2018	Forest Ecology and Management	Dynamics of detrital carbon pools following harvesting of a humid eastern Canadian balsam fir boreal forest	Boreal coniferous
Yang	2018	Biogeosciences	Dynamics of Post-fire Aboveground Carbon in A Chronosequence of Chinese Boreal Larch Forests	Boreal coniferous
Wang	2019	Forests	Nutrient Allocation to Different Compartments of Age-Sequence Larch Plantations in China	Boreal coniferous
Zhang	2019	Forests	Biomass Accumulation and Carbon Sequestration in an Age-Sequence of Mongolian Pine Plantations in Horqin Sandy Land, China	Boreal coniferous

Supplementary References

1. Hurtt, G. C. *et al.* Harmonization of Global Land-Use Change and Management for the Period 850–2100 (LUH2) for CMIP6. *Geoscientific Model Development Discussions* 1–65 (2020) doi:https://doi.org/10.5194/gmd-2019-360.
2. Friedlingstein, P. *et al.* Global Carbon Budget 2019. *Earth System Science Data* **11**, 1783–1838 (2019).
3. Klein Goldewijk, K., Beusen, A., Doelman, J. & Stehfest, E. Anthropogenic land use estimates for the Holocene – HYDE 3.2. *Earth System Science Data* **9**, 927–953 (2017).
4. Hansen, M. C. *et al.* High-resolution global maps of 21st-century forest cover change. *science* **342**, 850–853 (2013).
5. Houghton, R. A. & Nassikas, A. A. Global and regional fluxes of carbon from land use and land cover change 1850–2015. *Global Biogeochem. Cycles* **31**, 2016GB005546 (2017).
6. Pugh, T. A. M. *et al.* Role of forest regrowth in global carbon sink dynamics. *Proc Natl Acad Sci USA* **116**, 4382 (2019).
7. Arneth, A. *et al.* Historical carbon dioxide emissions caused by land-use changes are possibly larger than assumed. *Nature Geosci* **10**, 79–84 (2017).
8. Yue, C., Ciais, P. & Li, W. Smaller global and regional carbon emissions from gross land use change when considering sub-grid secondary land cohorts in a global dynamic vegetation model. *Biogeosciences* **15**, 1185–1201 (2018).
9. Mertz, O. *et al.* A fresh look at shifting cultivation: Fallow length an uncertain indicator of productivity. *Agricultural Systems* **96**, 75–84 (2008).
10. Koch, A., Brierley, C., Maslin, M. M. & Lewis, S. L. Earth system impacts of the European arrival and Great Dying in the Americas after 1492. *Quaternary Science Reviews* **207**, 13–36 (2019).
11. Hurtt, G. C. *et al.* The underpinnings of land-use history: three centuries of global gridded land-use transitions, wood-harvest activity, and resulting secondary lands. *Global Change Biology* **12**, 1208–1229 (2006).
12. Avitabile, V. *et al.* An integrated pan-tropical biomass map using multiple reference datasets. *Glob Change Biol* **22**, 1406–1420 (2016).
13. Liu, Y. Y. *et al.* Recent reversal in loss of global terrestrial biomass. *Nature Clim Change* **5**, 470–474 (2015).
