# Supplementary material for: Contribution of land use to the interannual variability of the land carbon cycle
Source: Nat Commun. 2020 Jun 23;11:3170. doi: 10.1038/s41467-020-16953-8 (PMC7311403; doi:10.1038/s41467-020-16953-8)
Supplement: Supplementary file 2 — Peer Review File [file 41467_2020_16953_MOESM2_ESM.docx]

Peer Review Information

**Journal:** Nature Communications

**Manuscript title:** Click here to enter text.

**Corresponding author name(s):** Click here to enter text.

**Editorial notes:**

**none**

**Reviewer comments & decisions:**

**Reviewer comments, first version:**

Reviewer #1 (Remarks to the Author):

The manuscript „Contribution of land use tot he interannual variability of the land carbon cycle“ presents a new assessment of gross C fluxes and their interannual variabilities on areas recovering from deforestation or wood harvest. The authors apply the ORCHIDEE model to intact and used lands separately and thus are able to assess the contribution of land use to terrestrial ecosystem C fluxes and their interannual variability. The study provides relevant insights into the impact of land-use on variabilities in the global C cycle, is well written and methodologically sound.
My recommendations for revisions concern mostly issues of data uncertainty and language.
In general, I encourage a more cautious discussion of data and methods uncertainties, and a discussion of the results‘ robustness (and lack of robustness). Specific issues include:
• How are the processes of Figure 1 implemented in the ORCHIDEE model? [The detailed description of potential processes following wood harvest / deforestation are mostly relevant in this context]. How do scale effects affect these processes in the model implementation (e.g., rotation periods in fairly small-scale shifting cultivation vs. Large-scale reforestation after several years of agricultural use)?
• How do you explain the variation between observed and calculated C stocks (Figures S4 and S5)? How do these differences affect your findings? Are some processes after reforestation better represented in the model than others? Some world regions better captured than others? Etc.
• Possibly my greatest concern regards your attribution of 100% primary forest (most of which Age group 6) in 1700 (Figure S7). While wood extraction was arguably much less intensive then, forests may very well have been substantially used in traditional agricultural practices (e.g., shifting cultivation in the tropics, forest grazing in temperate and boreal regions). The effect of such practices in 1700 on global tree stand age (or biomass density) is currently not quantified and beyond the scope of your study. However, the effect of this methodological choice on the model spin-up and the final results should be discussed, and a sensitivity analysis should be performed.
The notion of „intact“ land is somewhat misleading. In Figure S6 you imply that all land continuously used in a particular way is intact (including cropland and pastures). „Intact forest“ on the other hand implies previously non-managed forest land. I urge for more clarity here, defining more clearly what land is used and unused. You may also consider revising Figure S6 to show the lines as stacked areas.


Reviewer #2 (Remarks to the Author):

Yue et al., Contribution of land use on the interannual variability of the land carbon cycle

The manuscript by Yue et al. presents a quantification of the contribution of land use and land use change to the interannual variability (IAV) of the net terrestrial sink and therewith to the IAV of the CO2 growth rate. For this, the CO2 fluxes related to land use and land use change simulated with the ORCHIDEE model were decomposed into components, and these individual components were assessed. Results from the modelling were compared with estimates derived from a bookkeeping method, and methodological differences between the modelling and the bookkeeping method are discussed.

This manuscript is an interesting contribution to ongoing efforts aiming to quantify and explain the terrestrial contribution to the global carbon budget, and I consider the model developments made here an important step forward to increase our understanding of this contribution. The manuscript is of interest to a wide audience and suitable for publication in Nature Communications. However, I have some concerns and questions, primarily related to the terms used in the separation and the representation of land use, which I think requires further clarification. I would like to share these concerns (as well as some minor remarks) below, hoping that these will improve the manuscript further.


Major remarks:

The approach chosen for quantifying the land use and land use change flux E_LUC has a very strong focus on land use change and tends to hide the fluxes related to land that is continuously under land use. This may be valid when discussing long-term changes in the budget, for which the difference between (primary or secondary) forest on the one hand and agricultural land on the other present the main difference, because of the large difference in the C pools between the two. However, when discussing IAV, I expect the “unchanged” agricultural land to play an important role as well. The dynamics of this “unchanged” land are now part of the E_legacy flux (I presume – please correct me if I am wrong, see also l. 261), and I would hence expect IAV of E_legacy to show similar dynamics as S_intact, i.e. with IAV being dominated by the temperature response, presumably through GPP (as might be expected for S_intact, see e.g. Wang et al., 2013 or Ahlström et al., 2015). Yet, the manuscript attributes IAV of E_legacy solely to decomposition of legacy soil C – is there no GPP or Reco component of the “unchanged” land in here?

In addition to this, land use change is discussed only as shifts between forest ecosystems and agricultural land. Again, this may be the most important factor for the long-term trend, but not necessarily for IAV. How important are shifts from non-forest ecosystems (savanna or steppe) to agriculture for IAV?

In particular, I miss any remarks or discussion about the role of irrigation. As it is not mentioned in the manuscript, I presume that ORCHIDEE does not account for it (if it does, please add this to the Methods section). And whereas it may be hard to quantify its importance if ORCHIDEE does not represent it, I think that it should at least be discussed: After all, a considerable part (~20%) of agricultural land is under some sort of irrigation, and I would expect irrigation to remove part of the IAV in the uptake of CO2 (by reducing the variability originating from IAV of precipitation).

All in all, my feeling is that the shifts presented in the computed balance and in Fig. 1 probably represent the most important processes for long-term trends, but not necessarily for IAV. If the role of “(intact) land use” on the one hand and “land use change” on the other could be separated, I would advocate that. However, the model setup may not allow for this, in which case I would suggest to highlight the land use contribution more.


Minor remarks:

l. 47: “denote” should read “denotes”
Figure 1 gives a nice overview of the processes and fluxes, but I the time axis, which seems to start in the middle of the figure and run in two directions, confused me.

l. 74: The statement on different IAVs probably needs more explanation. What causes IAV in fuel wood collection to be different? Do you use a data product that quantifies annual fuel wood collection? And if the emissions appear at the year of harvest (which I understand as burning the C entirely in one year), what causes the “yearly to decadal” time scales that are referred to?

l. 111: “than in” should read “as in”

Figure 2: What is the unit of the colour scale here? It is given as Tg C yr-1, but it ought to contain a spatial dimension (per gridcell?). I would suggest to present it per unit of space (m-2, ha-1, or similar).

Figure 3: I find the difference in scaling between the positive and the negative axes in this figure very confusing – intuitively, one tries to add up positive and negative contributions “by eye” to obtain the black line, and this is now impossible. I would strongly recommend using the same scaling in positive and negative direction.

l. 254: “explains” should read “explain”

l. 285: “put question on” should read “call into question”, or similar

Supp. Fig. S4: “the decade of the 2000s”: Is this 2000-2009, or 2001-2010? Please specify.

Supp. Fig. S6: “intact” cropland and pasture are not part of S_intact, but of E_LUC, right? The use of “intact” confused me here.


References

Ahlström, A., Raupach, M. R., Schurgers, G., Smith, B., Arneth, A., Jung, M., … Zeng, N. (2015). The dominant role of semi-arid ecosystems in the trend and variability of the land CO2 sink. Science, 348(6237). https://doi.org/10.1126/science.aaa1668

Wang, W., Ciais, P., Nemani, R. R., Canadell, J. G., Piao, S., Sitch, S., … Myneni, R. B. (2013). Variations in atmospheric CO2 growth rates coupled with tropical temperature. Proceedings of the National Academy of Sciences of the United States of America, 110(32), 13061–13066. https://doi.org/10.1073/pnas.1219683110


Reviewer #3 (Remarks to the Author):

Contribution of land use to the interannual variability of the land carbon cycle, by Yue et al., is an interesting paper that demonstrates that the interannual variability of managed and disturbed ecosystems exceeds that of intact ecosystems. They use a novel configuration of the Orchidee DGVM that accounts for managed and unmanaged tiles within a model gridcell separately, to demonstrate that a bookkeeping approach underestimates the IAV of carbon fluxes due to land use change, and that when this IAV is improperly attributed to the residual land sink, it overestimates the climate sensitivity of intact forests. In particular, the legacy carbon flux and the recovery flux are more prone to climate-driven variability than a bookkeeping approach suggests.

The methods indicate close collaboration between ecosystem modelers and authors of a benchmark study using the bookkeeping approach to ensure that the boundary conditions for land use used by the model was consistent and comparable to published results. The authors do a nice job laying out the various fluxes that contribute to ELUC in Figure 1. Figures 3 and 4 provide a nice side-by-side comparison of the model and bookkeeping approach. I did have a question about each of them. In Fig. 3, Orchidee simulates a smaller Srecovery than is accounted for in the bookkeeping approach. Can the authors explain this or link this as a consequence of the enhanced climate sensitivity of recovering ecosystems? In Fig. 4, panel b appears to suggest that there is more IAV at the atmosphere, ocean interface, but this is not commented on in the paper. Is this just a visual illusion? If so, perhaps putting ocean as the top flux in the sinks, rather than the bottom flux in the sinks, would be a way of avoiding this visual illusion. If this enhanced variability in atmosphere-ocean exchange is real, does it indicate that the increased IAV in LUC fluxes requires, as a mass balance consequence, more variability in the ocean uptake?

One major comment I had was that it would be nice to see the coherence between the overall IAV, the ELUC IAV, and IAV in the atmospheric CO2 growth rate, since ultimately this observation provides a top-down constraint on interannual variations in surface fluxes. Atmospheric CO2 folds in IAV from fossil and ocean fluxes, but nevertheless, the argument that the paper makes would be better substantiated by showing that the model accounts for variations in atmos9pheric CO2.

I would have liked to see more validation in the main body of the paper. The paper itself only mentions validation on line 112, and then a bit more in the methods section at the end. One other area where this comes up is in the Efire, where variability seems low. The paper should potentially compare the modeled Efire against something like GFED, and explain the ramifications of any differences on their estimate of the role of managed vs unmanaged lands and the climate sensitivity thereof.

As more minor comments, Fig. 2 should have labels on the color bars. Fig. 5 should have the flux indicated on the y-axis, not in the upper left-hand corner of each panel.

Figs 1-5 are relatively blurry, whereas Fig. 6 looks very crisp. Whatever graphics rendering was used for Fig. 6 should be used throughout for the final publication.

Line 77 “is the composite term of these component fluxes” – could this be stated more simply as “is the sum of these component fluxes”??

Line 84 What is “it” in the subject?

Line 100 “it’s necessary DGVM’s capabilities” ◊ “it is necessary that the capabilities of DGVMs be expanded”

**Author rebuttal, first version:**

**Reviewer #1 (Remarks to the Author):**

4

5	The manuscript “Contribution of land use to the interannual variability of the land carbon cycle”

6	presents a new assessment of gross C fluxes and their interannual variabilities on areas recovering

7	from deforestation or wood harvest. The authors apply the ORCHIDEE model to intact and used lands

8	separately and thus are able to assess the contribution of land use to terrestrial ecosystem C fluxes

9	and their interannual variability. The study provides relevant insights into the impact of land-use on

10	variabilities in the global C cycle, is well written and methodologically sound.

11

12	My recommendations for revisions concern mostly issues of data uncertainty and language.

13	In general, I encourage a more cautious discussion of data and methods uncertainties, and a

14	discussion of the results‘ robustness (and lack of robustness).

15

16	We appreciate the reviewer’s efforts and thank the reviewer for the general positive comments. As

17	also part of the responses to the comments by other reviewers, the improvements on data/methods

18	and results uncertainties include: (1) We discussed the influences on the IAV of _ELUC_ by the temporal

19	resolutions of the LUC forcing data (forest area changes and wood harvest) and their implementation

20	in both bookkeeping models and ORCHIDEE DGVM (lines 239-253). (2) We added a new section in

21	Supplementary Material entitled ‘Model evaluation and comparisons with observations’ to enhance

22	the validation, where model evaluations of simulated biomass density were expanded to incorporate

23	more observations. The influences of model error on simulated _ELUC_ and other carbon fluxes were

24	discussed in the main text. (3) We added comparisons of simulated intact and secondary forest sinks

25	with forest inventory-based observations (lines 183-199). (4) We performed a sensitivity model run

26	that included local-scale shifting cultivation starting from 1500 (the SC-sensitivity run) on top of the

27	baseline simulation presented in the original manuscript. (5) The influence of the lack of cropland

28	irrigation on the results was discussed (lines 348-353).

29

30	The language of the manuscript has been improved by a native English speaker and editor (Dr. David

31	Nielsen, [https://www.researchgate.net/profile/DC_Nielsen/contributions](https://www.researchgate.net/profile/DC_Nielsen/contributions))). Please find below our

32	point-by-point responses to the reviewer comments. All revised texts in the main manuscript and

33	Supplementary Material are marked in blue.

34

35	Specific issues include:

36

37	[1] How are the processes of Figure 1 implemented in the ORCHIDEE model? [The detailed

38	description of potential processes following wood harvest / deforestation are mostly relevant in this

39	context]. How do scale effects affect these processes in the model implementation (e.g., rotation

40	periods in fairly small-scale shifting cultivation vs. Large-scale reforestation after several years of

41	agricultural use)?

42

43	In response to the reviewer’s comments, we have added two paragraphs (lines 551-579) in the

44	Methods section describing the detailed implementation of land use change processes in

45	combination with the cohort functionality in ORCHIDEE. To ensure full comparability with the

46	HN2017 study, we used the same parameterizations to describe the fate and transitions of different

47	carbon pools when a land use transition occurs. Detailed descriptions for such parameterizations are

48	provided in Supplementary Table S1.

49

50	The LUC implementation in ORCHIDEE per se does not generate any extra scale effects on LUC

51	processes. ORCHIDEE explicitly accounts for all possible transitions between vegetation types,

52	including simultaneous deforestation and re/afforestation within a single grid cell. Regardless of

53	their original spatial resolution, land transitions in the input LUC forcing data can always be summed

54	and re-implemented over ORCHIDEE model grids. The scale-related uncertainty of LUC areas is thus

55	independent of the model spatial resolution but depends on the original forcing data. Nonetheless,

56	we argue that net land use change (i.e., deforestation, afforestation, and reforestation) and wood

57	harvest largely reflect large-scale processes, while shifting cultivation largely reflects local-scale

58	process. We added Section 6 in the Supplementary Material to discuss the scale issues, and briefly

59	summarized them in the main text lines 105-107 and lines 116-119, Methods section lines 566-569,

60	lines 584-586, and lines 596-598.

61

62	[2] How do you explain the variation between observed and calculated C stocks (Figures S4 and S5)?

63	How do these differences affect your findings? Are some processes after reforestation better

64	represented in the model than others? Some world regions better captured than others? Etc.

65

66	A more thorough model validation has been made in the revised manuscript, but due to the length

67	limitation of the main text, model validation and relevant discussions were split into main text and

68	the Supplementary Material. Major changes include the following:

69

70	- In the Supplementary Material, a new section entitled “Model evaluation and comparisons

71	with observations” has been added, which includes as the authors’ revision efforts: (1)

72	Multiple satellite-based forest aboveground biomass observations were used to enhance the

73	validation of simulated spatial distribution of forest aboveground biomass (Supplementary

74	Fig. S5). Comparisons of simulated vs. observed biomass were made for each grid cell and

75	detailed discussions of regional over- or underestimations were discussed. (2) We compiled a

76	new database of forest biomass growth using literature-based forest chronosequence studies

77	that cover boreal, temperate, and tropical climates with distinctions being made between

78	broadleaf and coniferous forests. Biomass-age growth patterns were compared for all three

79	biomes (Supplementary Fig. S4, Table S2). Insights from evaluations were used to explain

80	model error in light of validation by satellite data.

81

82	- In the main text, we added: (3) Carbon sinks over forests simulated by ORCHIDEE were

83	compared with inventory-based observations for different regions (main text Fig. 3). These

84	validation efforts, along with other validations that were already in the original manuscript,

85	were now summarized in a paragraph in the Introduction of the main text (lines 129-140).

86

87	Following these model validations, model errors and potential causes, as well as their influences on

88	the result uncertainties were discussed. Please refer to the following sections in the revised

89	manuscript: lines 163-166 of the main text where the influences on estimated _ELUC_ ^and^ _Snet_ were

90	discussed; lines 197-199 of the main text, where errors in simulated forest carbon sink were

91	discussed. But in general, detailed discussions of model error on regional basis were only presented

92	in the Supplementary Material due to the length limitation of the main text.

93

94	Forest-relevant carbon fluxes (GPP, NPP, heterotrophic respiration, etc.) and pools (aboveground,

95	belowground, litter, etc.) after reforestation were results of complex modeling chains ranging from

96	photosynthesis, autotrophic respiration, allocation, turnover to mortality in the model. We mainly

97	focused on validating processes and variables that are relevant to our research purpose in this study.

98	Detailed discussions on which processes leading to which specific errors are considered beyond the

99	scope here and perhaps overwhelming for a study with a global focus.

100

101	[3] Possibly my greatest concern regards your attribution of 100% primary forest (most of which Age

102	group 6) in 1700 (Figure S7). While wood extraction was arguably much less intensive then, forests

103	may very well have been substantially used in traditional agricultural practices (e.g., shifting

104	cultivation in the tropics, forest grazing in temperate and boreal regions). The effect of such practices

105	in 1700 on global tree stand age (or biomass density) is currently not quantified and beyond the

106	scope of your study. However, the effect of this methodological choice on the model spin-up and the

107	final results should be discussed, and a sensitivity analysis should be performed.

108

109	We agree with the reviewer that agricultural practices such as shifting cultivation or forest grazing

110	would have an impact on 1700 global forest biomass and age structure. We did not include shifting

111	cultivation in our first draft mainly because we would like to ensure the same boundary conditions

112	(i.e., LUC processes and their areas) in the ORCHIDEE simulation and the HN2017 bookkeeping

113	model, in order to highlight the influences of these two approaches on the attribution of IAV of Snet

114	(despite the fact that some earlier versions of HN2017 did include shifting cultivation, e.g., that used

115	in Pan et al., 2011). The second reason for not including shifting cultivation is the great uncertainty in

116	its areas and historical changes (Houghton and Nassikas, 2018). These points are now included in the

117	revised main text lines 102-107 and lines 117-123.

118

119	In the revised manuscript, we tested the sensitivity of results by including pre-1700 LUC activities by

120	performing a sensitivity model run accounting for shifting cultivation (the SC-sensitivity run) starting

121	from 1500. The inclusion of forest grazing was restricted by both forcing data availability and the lack

122	of its representation in the model, and was thus not included. The advantage of including only

123	shifting cultivation, on top of net land use change and wood harvest included in the baseline

124	simulation, is that the additional effects of shifting cultivation can be diagnosed by differences in the

125	two simulations. The areas undergoing shifting cultivation used in the SC-sensitivity run were

126	extracted from a widely used gridded LUC dataset of LUH2. We added in Supplementary Material

127	Section 5 for detailed descriptions of the setup of the SC-sensitivity run and preparation of the

128	forcing data. A summary of the SC-sensitivity run was provided in the Methods section (lines 596-

129	606), and in the Introduction section of the revised manuscript (lines 123-125).

130

131	Figure R1 (below) shows the model behaviour and the influence of pre-1700 shifting cultivation on

132	the 1700 forest age structure. There was a sharp decrease in the intact forest area (Fig. R1a) and

133	increase of young forest cohorts (Age 1 to Age 3, Fig. R1b) during the initial 20 years after the

134	inception of shifting cultivation in 1500. The decrease of intact forests was due to land clearing, while

135	the increase of young forests was caused by agricultural fallow. Afterwards both areas of intact forest

136	and young forest cohort stabilized, when a stable rotation cycle in shifting cultivation was

137	established. In the ORCHIDEE implementation of shifting cultivation, clearing of forest starts in

138	priority from the Age 3 cohort, roughly corresponding to a forest age of 20 years in the tropics. This

139	rotation length used in the model is in agreement with documented fallow length in the literature

140	(Hurtt et al., 2006; Koch et al., 2019; Mertz et al., 2008). As shown in Figure R1, it’s clear that at the

141	year of 1700 the SC-sensitivity run results in lower intact forest area and higher secondary forest area

142	than the baseline simulation.

143

144

145	Figure R1. Dynamics of areas for (a) intact forest and (b) secondary forest cohorts for 1500-1700

146	simulated by the SC-sensitivity run.

147

148	We further compared various variables between the two simulations, including the areas of

149	secondary forest, secondary grassland, and post-1700 agricultural land (Supplementary Figure S12),

150	carbon fluxes of ELUC, Snet, ^and^ _SIntact_ (Supplementary Figure S14), and fluxes of ELUC, Efire, Ewood, Elegacy

151	and _Srecov_ (Supplementary Figure S13).

152

153	In general, including shifting cultivation results in more managed lands of all types and increased all

154	component fluxes of _ELUC_ except for _Ewood._ Shifting cultivation led to further increases in _ELUC_ because

155	the increase in gross emissions offset the increase in gross sinks by secondary forests. Because of

156	lower intact forest area after including shifting cultivation, the carbon sink by intact lands was lower.

157	Collectively, a higher _ELUC_ and lower _SIntact_ leads to a smaller simulated net land carbon sink _(Snet)._ The

158	contribution of managed land to the IAV of _Snet_ has increased and the temperature sensitivity of SIntact

159	was further reduced. All Results sections considering SC-sensitivity runs are discussed in the main

160	text (lines 317-332, 344-345).

161

162	[4] The notion of „intact“ land is somewhat misleading. In Figure S6 you imply that all land

163	continuously used in a particular way is intact (including cropland and pastures). „Intact forest“ on

164	the other hand implies previously non-managed forest land. I urge for more clarity here, defining

165	more clearly what land is used and unused. You may also consider revising Figure S6 to show the

166	lines as stacked areas.

167

168	To enhance the clarity, we now use the terms of ‘intact’ forest and ‘intact’ grassland exclusively for

169	forest and grassland that have not been ‘touched’ by human land use and management since 1700

170	(note that for forest, ‘intact’ forests also includes forests having recovered for a sufficiently long time

171	and reaching a similar status as intact ones). We use ‘permanent agricultural land’ or ‘pre-1700

172	agricultural land’ for agricultural lands established prior to 1700, and ‘post-1700 agricultural land’ for

173	those established after 1700. Both types of agricultural land, along with the secondary grassland and

174	forest, are ‘managed land’ and carbon fluxes over all sorts of managed land were included in the ELUC

175	in the revised manuscript.

176

177	There is a small mismatch between the _ELUC_ definition here and that in HN2017, as the latter does not

178	include contributions from ‘permanent agricultural land’. However, Supplementary Figure S10 shows

179	that the contribution of this flux is rather small with low IAV because the carbon balance of

180	‘permanent agricultural land’ stabilized a long time ago, and therefore its influence on our ELUC

181	compared with HN2017 is negligible. We acknowledge the fact that a change of management

182	intensity over ‘permanent agricultural land’ may still cause changes in its carbon balance, for good

183	(e.g., cover crops, reduced tillage, conservation agriculture) or for bad (e.g., increased tillage,

184	increased erosion, degradation). These processes belonging to a broad category of land management

185	without land use change were not included in our study.

186

187	The new definition of _ELUC_ in ORCHIDEE and its influence on the _ELUC_ are presented in the revised

188	manuscript lines 644-649 (Methods section), lines 73-75 in the Introduction section, and lines 224-

189	229 in the Results section in the main text.

190

191	We revised Supplementary Figure S6 as a stacked area plot as suggested by the reviewer.

192

193

194	**Reviewer #2 (Remarks to the Author):**

195

196	Yue et al., Contribution of land use on the interannual variability of the land carbon cycle

197

198	The manuscript by Yue et al. presents a quantification of the contribution of land use and land use

199	change to the interannual variability (IAV) of the net terrestrial sink and therewith to the IAV of the

200	CO2 growth rate. For this, the CO2 fluxes related to land use and land use change simulated with the

201	ORCHIDEE model were decomposed into components, and these individual components were

202	assessed. Results from the modelling were compared with estimates derived from a bookkeeping

203	method, and methodological differences between the modelling and the bookkeeping method are

204	discussed.

205

206	This manuscript is an interesting contribution to ongoing efforts aiming to quantify and explain the

207	terrestrial contribution to the global carbon budget, and I consider the model developments made

208	here an important step forward to increase our understanding of this contribution. The manuscript is

209	of interest to a wide audience and suitable for publication in Nature Communications. However, I

210	have some concerns and questions, primarily related to the terms used in the separation and the

211	representation of land use, which I think requires further clarification. I would like to share these

212	concerns (as well as some minor remarks) below, hoping that these will improve the manuscript

213	further.

214

215	We appreciate the reviewer’s efforts to review our paper. We have clarified the concepts related to

216	‘permanent agricultural land’ established prior to 1700 and those established after 1700.

217	Representations of land use change in the model are also further clarified. Please find below our

218	point-by-point responses to the reviewer’s comments. All revised texts in the main manuscript and

219	Supplementary Material are marked in blue.

220

221	Major remarks:

222

223	[1] The approach chosen for quantifying the land use and land use change flux E_LUC has a very

224	strong focus on land use change and tends to hide the fluxes related to land that is continuously

225	under land use. This may be valid when discussing long-term changes in the budget, for which the

226	difference between (primary or secondary) forest on the one hand and agricultural land on the other

227	present the main difference, because of the large difference in the C pools between the two.

228	However, when discussing IAV, I expect the “unchanged” agricultural land to play an important role

229	as well. The dynamics of this “unchanged” land are now part of the E_legacy flux (I presume – please

230	correct me if I am wrong, see also l. 261), and I would hence expect IAV of E_legacy to show similar

231	dynamics as S_intact, i.e. with IAV being dominated by the temperature response, presumably

232	through GPP (as might be expected for S_intact, see e.g. Wang et al., 2013 or Ahlström et al., 2015).

233	Yet, the manuscript attributes IAV of E_legacy solely to decomposition of legacy soil C – is there no

234	GPP or Reco component of the “unchanged” land in here?

235

236	We acknowledge that our previous approach was potentially confusing where we included the

237	agricultural lands established before 1700 (‘permanent agricultural land’) as part of ‘intact’ land and

238	not as part of _ELUC._ This point was also raised by Reviewer #1 (please refer to Major Comment #4 by

239	the first reviewer and our responses there), and raised somewhat again in last major comment by

240	Reviewer #2.

241

242	In response to the reviewer’s comments and for the sake of scope coherence to include in _ELUC_ all

243	managed lands, we included carbon fluxes over permanent agricultural land into _ELUC_ in the revised

244	manuscript. This new scope of _ELUC_ differs slightly with that used by HN2017 and by the DGVMs

245	participating in the GCP annual carbon budget updates. However, we found that the influence of this

246	scope difference was negligible when it comes to the comparability of _ELUC_ estimates between

247	ORCHIDEE and HN2017. This was because net carbon fluxes over permanent agricultural land are a

248	very small sink with much lower IAV, while those over post-1700 agricultural land were a large source

249	with much higher IAV (Supplementary Figure S10, please also refer to our reply to Comment #4 of

250	the first Reviewer).

251

252	The revisions relating to the new definition of _ELUC_ in ORCHIDEE and its influence on the result

253	uncertainties can be found in the revised manuscript lines 644-649 (Methods section), lines 73-75 in

254	the Introduction section, and lines 224-229 in the Results section in the main text.

255

256	The reviewer is correct that _Elegacy_ is expected to show similar dynamics as _SIntact._ As is shown in Figure

257	R2a (below), _Elegacy_ shows a significant positive sensitivity to the tropical air temperature anomaly (T).

258	This is consistent with a negative T sensitivity of _SIntact_ given the sign conventions (main text Figure

259	7b). As is expected and congruent with the studies cited by the reviewer, simulated GPP over post-

260	1700 agricultural lands shows a negative relationship with T (Figure R2b), but the R^2^ is much lower

261	than that of _Elegacy._ We further examined the temperature sensitivities of the net carbon input into

262	soil and litter _(Cinput,_ being NPP subtracted by crop and pasture harvest) and heterotrophic respiration

263	(Rh) (Figure R2c and R2d). _Cinput_ is negatively correlated to temperature with a higher R^2^ than GPP,

264	indicating its more direct relevance to _Elegacy._ Rh has a positive correlation with T. These correlation

265	relationships are consistent with our current understanding of drivers of IAV of net carbon fluxes in

266	response to climate variations. But in ORCHIDEE, it seems that the temperature sensitivity of Elegacy

267	was driven by at least both sensitivities of _Cinput_ and Rh and less directly by that of GPP.

268

269

270	Figure R2. Temperate sensitivities of different carbon fluxes over agricultural land (including both

271	pre- and post-1700 ones).

272

273	We agree with the reviewer that _Elegacy_ (defined as the reverse of NBP over agricultural land in

274	ORCHIDEE simulation) contains GPP and _Reco_ (ecosystem respiration) components. As _Reco_ contains

275	autotrophic and heterotrophic respiration, while autotrophic respiration could be canceled from GPP

276	when considering _Cinput,_ we here consider only _Cinput_ and Rh as the two gross flux components

277	governing the IAV of _Elegacy._ We assume that the IAV of _Elegacy_ is dominated by Rh of slash and soil

278	carbon from past LUC activities, but this cannot be directly diagnosed from ORCHIDEE, because LUC-

279	derived slash and soil C were mixed with input from agricultural straw in the model. To partly

280	demonstrate this, we examined the dynamics of _Elegacy_ resulting from shifting cultivation only,

281	quantified as the difference in _Elegacy_ ( _Elegacy)_ between the SC-sensitivity run accounting for shifting

282	cultivation and the baseline run.

283

284	Figure R3 shows the dynamics of Elegacy, Cinput, and Rh for 1900-2015. When the shifting cultivation

285	area increased sharply around 1940 and peaked around 1990 (Supplementary Figure S11), all three

286	fluxes show corresponding increases starting at 1940 (Figure R3). The increase in _Cinput_ is driven by

287	increases in agricultural land area. But the increase in Rh is much steeper than _Cinput,_ which must be

288	driven not only by increased agricultural straw input but also by LUC-derived slash and soil C. More

289	importantly, the temporal pattern of _Elegacy_ generally follows that of Rh rather than _Cinput,_ partly

290	supporting our educated guess that Rh resulting from slash and soil C decomposition dominates the

291	IAV of Elegacy.

292

293

294	Figure R3. The effects of shifting cultivation on the dynamics of _Cinput,_ Rh, and _Elegacy._ The differences in

295	fluxes of _Cinput,_ Rh and _Elegacy_ between the SC-sensitivity and baseline runs were shown as _ΔCinput,_ ΔRh

296	and ΔElegacy.

297

298	The other evidence to support our assumption that newly established agricultural land is dominated

299	by slash and soil C decomposition is Supplementary Figure S10. The post-1700 agricultural lands

300	show a strong source term for 1959-2015, but pre-1700 so-called ‘permanent agricultural lands’

301	show a small sink term with very low IAV, indicating that in the former case both IAV and magnitude

302	of carbon fluxes in post-1700 agricultural land must be dominated by LUC-derived legacy and soil C

303	input rather by the GPP of the agricultural land itself.

304

305	These results support our inference made in the Methods section that ‘NBP over recently established

306	agricultural land was dominated by the decomposition of legacy slash and soil carbon originating

307	from former intact land’.

308

309	[2] In addition to this, land use change is discussed only as shifts between forest ecosystems and

310	agricultural land. Again, this may be the most important factor for the long-term trend, but not

311	necessarily for IAV. How important are shifts from non-forest ecosystems (savanna or steppe) to

312	agriculture for IAV?

313

314	In Figure 1 we used forest-cropland transitions as examples to explain the LUC processes simulated in

315	ORCHIDEE. Transitions between natural grassland and agricultural land were also included in our

316	simulation. This is explained in the revised manuscript lines 45-47, lines 105-107, lines 112-113 in the

317	main text, lines 561-566 and lines 583-586 in the Methods section, and Supplementary Figure S6a.

318	The caption of Figure 1 has also been revised to include the grassland-related transitions (please

319	refer to the revised caption for Fig. 1). Transitions between savanna and agricultural land, which are

320	typical in tropics, were also included. But because ORCHIDEE does not have a savanna functional

321	type, savanna areas have been disintegrated into forest and grassland. This is explained in the

322	revised manuscript lines 564-566 (the Methods section).

323

324	In the revised manuscript, we further included shifting cultivation between these two vegetation

325	types (forest and natural grassland) and agricultural land in an SC-sensitivity run. This is described in

326	the revised Methods lines 596-606 and Supplementary Material Section 5.

327

328	In summary, all component fluxes of _ELUC_ in our study thus included both conversions of forest as well

329	as grassland ecosystem. As shown in Supplementary Figure S6, Figure S11, and Figure S12, large

330	tracts of post-1700 agricultural lands were made from grassland by both net land use change and

331	shifting cultivation. However, we did not quantify the exact contribution by grassland-agricultural

332	transitions only, as this would require extra factorial simulations and was not the central focus of our

333	paper.

334

335	[3] In particular, I miss any remarks or discussion about the role of irrigation. As it is not mentioned in

336	the manuscript, I presume that ORCHIDEE does not account for it (if it does, please add this to the

337	Methods section). And whereas it may be hard to quantify its importance if ORCHIDEE does not

338	represent it, I think that it should at least be discussed: After all, a considerable part (~20%) of

339	agricultural land is under some sort of irrigation, and I would expect irrigation to remove part of the

340	IAV in the uptake of CO2 (by reducing the variability originating from IAV of precipitation).

341

342	We thank the reviewer for pointing this out. Irrigation in agriculture is not represented in this version

343	of ORCHIDEE and therefore not included in our study. We added the following sentences in the

344	revised manuscript to discuss the implications on our results (lines 348-353 in the main text): “On the

345	other hand, cropland irrigation was not simulated, but it may affect the IAV of cropland carbon

346	fluxes. Studies at county scale in the central US (Doughty et al., 2018) and continental scale over

347	Europe (Smith et al., 2010) demonstrated that irrigation relieves water stress and the associated

348	productivity drop over extreme drought periods. Irrigated croplands therefore may have a lower IAV

349	in carbon uptake than rainfed croplands. Because 17% of global cropland was irrigated as of 2015,

350	neglecting irrigation processes in our model might lead to overestimation of the IAV of ELUC.”.

351

352	[4] All in all, my feeling is that the shifts presented in the computed balance and in Fig. 1 probably

353	represent the most important processes for long-term trends, but not necessarily for IAV. If the role

354	of “(intact) land use” on the one hand and “land use change” on the other could be separated, I

355	would advocate that. However, the model setup may not allow for this, in which case I would suggest

356	to highlight the land use contribution more.

357

358	In relation to our reply to Comment #2, we revised the caption of Figure 1 to explain all LUC

359	processes included in our study. The role of ‘intact land use’ (i.e., permanent pre-1700 agricultural

360	land) and ‘land use change’ (i.e., post-1700 agricultural land) were also separated (Supplementary

361	Figure S10). Lines 224-229 in the main text presented these results.

362

363

364	Minor remarks:

365

366	[5] l. 47: “denote” should read “denotes”

367	Figure 1 gives a nice overview of the processes and fluxes, but I the time axis, which seems to start in

368	the middle of the figure and run in two directions, confused me.

369

370	We changed ‘denote’ to ‘denotes’. We revised Figure 1 to show all LUC processes and used only a

371	rightward direction to represent the evolution of land use. The caption of Figure 1 was also revised to

372	include all LUC processes in our study.

373

374	[6] l. 74: The statement on different IAVs probably needs more explanation. What causes IAV in fuel

375	wood collection to be different? Do you use a data product that quantifies annual fuel wood

376	collection? And if the emissions appear at the year of harvest (which I understand as burning the C

377	entirely in one year), what causes the “yearly to decadal” time scales that are referred to?

378

379	Both fuel and industrial wood harvest biomass data were derived from annual harvest volumes as

380	reported by the FAOSTAT for the period of 1961–2015. Before this period annual harvest data were

381	derived from historical reviews. The low IAV in annual wood harvest might be driven only by slow

382	changes in economic situations. These points are now discussed in the revised manuscript lines 246-

383	253.

384

385	[7] l. 111: “than in” should read “as in”

386

387	We changed the text as proposed by the reviewer.

388

389	[8] Figure 2: What is the unit of the colour scale here? It is given as Tg C yr-1, but it ought to contain a

390	spatial dimension (per gridcell?). I would suggest to present it per unit of space (m-2, ha-1, or

391	similar).

392

393	We revised Figure 2 to have the unit of g C m^-2^ yr^-1^ in terms of land area.

394

395	[9] Figure 3: I find the difference in scaling between the positive and the negative axes in this figure

396	very confusing – intuitively, one tries to add up positive and negative contributions “by eye” to

397	obtain the black line, and this is now impossible. I would strongly recommend using the same scaling

398	in positive and negative direction.

399

400	Figure 3 (now Figure 4 in the revised manuscript) has been revised to have the same scaling over

401	both vertical axes.

402

403	[10] l. 254: “explains” should read “explain”

404

405	We have changed the text as proposed by the reviewer.

406

407	[11] l. 285: “put question on” should read “call into question”, or similar

408

409	We changed the text as proposed by the reviewer.

410

411	[12] Supp. Fig. S4: “the decade of the 2000s”: Is this 2000-2009, or 2001-2010? Please specify.

412

413	This figure has been revised to include more satellite observations. The time period for each data has

414	been specified in the revised figure.

415

416	[13] Supp. Fig. S6: “intact” cropland and pasture are not part of S_intact, but of E_LUC, right? The use

417	of “intact” confused me here.

418

419	In the original manuscript they were part of _SIntact._ They were excluded from _ELUC_ mainly to follow the

420	_ELUC_ definition used in the bookkeeping models and in the DGVMs used in the GCP annual carbon

421	budgets. In the revised manuscript they were included in _ELUC._ The concept of _ELUC_ in the revised

422	manuscript includes carbon fluxes over all managed lands, including agricultural lands that were

423	established after 1700 (the start year of the simulation) and that were permanently used before

424	1700. Correspondingly, _SIntact_ includes carbon fluxes over intact forest and grassland only. These

425	changes are explained in the Methods section lines 638-639 and lines 644-649 and Results section

426	lines 224-229.

427

428

429	References

430

431	Ahlström, A., Raupach, M. R., Schurgers, G., Smith, B., Arneth, A., Jung, M., Zeng, N. (2015). The

432	dominant role of semi-arid ecosystems in the trend and variability of the land CO2 sink. Science,

433	348(6237). <https://doi.org/10.1126/science.aaa1668>

434

435	Wang, W., Ciais, P., Nemani, R. R., Canadell, J. G., Piao, S., Sitch, S., Myneni, R. B. (2013). Variations

436	in atmospheric CO2 growth rates coupled with tropical temperature. Proceedings of the National

437	Academy of Sciences of the United States of America, 110(32), 13061–

438	13066. <https://doi.org/10.1073/pnas.1219683110>

439

440	**Reviewer #3 (Remarks to the Author):**

441

442	Contribution of land use to the interannual variability of the land carbon cycle, by Yue et al., is an

443	interesting paper that demonstrates that the interannual variability of managed and disturbed

444	ecosystems exceeds that of intact ecosystems. They use a novel configuration of the Orchidee DGVM

445	that accounts for managed and unmanaged tiles within a model gridcell separately, to demonstrate

446	that a bookkeeping approach underestimates the IAV of carbon fluxes due to land use change, and

447	that when this IAV is improperly attributed to the residual land sink, it overestimates the climate

448	sensitivity of intact forests. In particular, the legacy carbon flux and the recovery flux are more prone

449	to climate-driven variability than a bookkeeping approach suggests.

450

451	The methods indicate close collaboration between ecosystem modelers and authors of a benchmark

452	study using the bookkeeping approach to ensure that the boundary conditions for land use used by

453	the model was consistent and comparable to published results. The authors do a nice job laying out

454	the various fluxes that contribute to ELUC in Figure 1. Figures 3 and 4 provide a nice side-by-side

455	comparison of the model and bookkeeping approach. I did have a question about each of them.

456

457	[1] In Fig. 3, Orchidee simulates a smaller Srecovery than is accounted for in the bookkeeping

458	approach. Can the authors explain this or link this as a consequence of the enhanced climate

459	sensitivity of recovering ecosystems?

460

461	We appreciate the reviewer’s efforts and express our thanks for the reviewer’s positive comments.

462	All revised text in the main manuscript and Supplementary Material is marked in blue.

463

464	The smaller _Srecov_ in ORCHIDEE than HN2017 can probably only be partly ascribed to the enhanced

465	climate sensitivity, if there was any, because under extreme circumstances such as drought, forest

466	biomass will suffer a loss so that it would take longer to recover after drought than in the case of a

467	static growth curve used in the bookkeeping approach.

468

469	Otherwise, the HN2017 bookkeeping approach might be overestimating global secondary forest sink.

470	The 1.5-2 Pg C yr^-1^ global secondary forest sink for 2000-2009 estimated by HN2017, when any forest

471	recovery from shifting cultivation was excluded, is higher than those reported by several DGVM-

472	based studies. The recent work by (Pugh et al., 2019) using satellite-derived forest age distribution

473	and the LPJ DGVM estimated global secondary forest sink as 0.53 Pg C yr^-1^ when environmental

474	changes such as CO2 concentration increase and N deposition are not accounted for, which is much

475	lower than HN2017. The estimate by HN2017 is also higher than earlier global secondary forest sink

476	estimates made by (Shevliakova et al., 2009) (2009; 0.35–0.6 Pg C yr^-1^ using the LM3V model) and

477	(Yang et al., 2010) (2010; 0.36 Pg C yr^-1^ using the ISAM-NC model).

478

479	Possible reasons for overestimation of secondary forest sink by the HN2017 bookkeeping model

480	include either higher static biomass carbon or soil organic carbon densities for different ecosystems

481	or shorter recovery times (typically 50 years for forest biomass carbon and 15–40 years for forest soil

482	organic carbon). For example, HN2017 used the biomass carbon density of 190 Mg C ha^-1^ (Table 1 in

483	Houghton and Nassikas, 2017), while most satellite-based observations show the aboveground

484	biomass carbon density for tropical rainforest as 100–140 Mg C ha^-1^ (Supplementary Figure S5), much

485	lower than that used in HN2017. Most of the static biomass and soil carbon densities used in the

486	bookkeeping model are based on observations for current times, which are likely higher than those

487	of forests under historical conditions with a lower CO2 concentration.

488

489	These points are discussed in the revised text lines 267-276.

490

491	[2] In Fig. 4, panel b appears to suggest that there is more IAV at the atmosphere, ocean interface,

492	but this is not commented on in the paper. Is this just a visual illusion? If so, perhaps putting ocean as

493	the top flux in the sinks, rather than the bottom flux in the sinks, would be a way of avoiding this

494	visual illusion. If this enhanced variability in atmosphere-ocean exchange is real, does it indicate that

495	the increased IAV in LUC fluxes requires, as a mass balance consequence, more variability in the

496	ocean uptake?

497

498	The seemingly higher IAV of the ocean CO2 uptake in the original Fig. 4b is an illusion by overlaying

499	the ocean sink on top of the temporally variable sinks of the land and the atmosphere. To avoid this

500	problem, we followed the reviewer’s suggestion to plot the ocean sink at the top of the sink fluxes.

501	Please refer to the revised Figure 5.

502

503	The second question raised by the reviewer (i.e., whether more IAV in LUC fluxes requires more IAV

504	in the ocean uptake) cannot be resolved within the scope of this study. In both revised Figures 5a and

505	5b we used the same ocean CO2 uptake data provided by the GCP, which was from an ensemble of

506	global ocean biogeochemistry models that meet observational constraints. This ocean uptake

507	estimate was treated as the ‘truth’ here. The net land CO2 uptake was taken as the residual and

508	further used to validate the ORCHIDEE model. Otherwise, the reviewer is correct that more IAV in the

509	ocean uptake must be needed, to offset the higher IAV in the sum of sink terms than in the source

510	terms (Figure 5b). But unfortunately, the higher IAV in the sum of all sink terms also includes the

511	error by ORCHIDEE to simulate the land sink, which could not be separated from the IAV of the ocean

512	uptake. Detailed discussion of the variability of the ocean uptake is beyond the scope in this study. In

513	general, the ocean uptake is thought to be better constrained than the land one, and deriving the net

514	land sink by the residual approach is widely accepted.

515

516	[3] One major comment I had was that it would be nice to see the coherence between the overall

517	IAV, the ELUC IAV, and IAV in the atmospheric CO2 growth rate, since ultimately this observation

518	provides a top-down constraint on interannual variations in surface fluxes. Atmospheric CO2 folds in

519	IAV from fossil and ocean fluxes, but nevertheless, the argument that the paper makes would be

520	better substantiated by showing that the model accounts for variations in atmospheric CO2.

521

522	As is explained above, the _Snet_ by the ‘bookkeeping and residual budget’ approach was actually

523	derived by a ‘top-down’ approach, where both IAVs from fossil emissions and ocean uptake were

524	taken into account. This observation-based _Snet_ is shown as the black line in the revised Figure 6a. The

525	_Snet_ simulated by ORCHIDEE was validated against the observation-based _Snet._ The ability by

526	ORCHIDEE to account for IAV in the atmospheric CO2 growth rates was thus accounted for in such

527	validation. Additionally, we also validated ORCHIDEE in terms of its ability to simulate the IAV of Snet

528	driven by the land temperature variations associated with ENSO climate oscillations (the revised

529	Figure 7a). The clarification of this issue is now included in the revised manuscript lines 137-140.

530

531	[4] I would have liked to see more validation in the main body of the paper. The paper itself only

532	mentions validation on line 112, and then a bit more in the methods section at the end.

533

534	In the revised manuscript, we provided one paragraph in the Introduction section (lines 129-140)

535	summarizing all validation efforts that have been made. In addition, a new paragraph has been

536	added in the main text Result section entitled “Spatial separation of net land carbon balance into

537	managed and intact lands” (lines 183-199) to present the model validation regarding simulated

538	global and regional forest carbon sinks.

539

540	Besides this, model validation has also been enhanced in general in the revised manuscript. A section

541	in the Supplementary Material entitled “Model evaluation and comparisons with observations” has

542	been added, where aboveground biomass data from ORCHIDEE were compared with multiple

543	satellite observations and forest biomass-age relationships were compared with a chronosequence-

544	based observation database compiled in this study for tropical, temperate and boreal forest biomes.

545	Please also refer to our responses to the Comment #2 of the first reviewer regarding the

546	improvements in validation in the revised manuscript.

547

548	Unfortunately, because of the length limit, we included in the main text only model validations that

549	are more directly relevant to the key message of our paper, and left the detailed descriptions of

550	model validation on biomass stocks in the Supplementary Material. We hope that this compromise

551	makes sense.

552

553	[5] One other area where this comes up is in the Efire, where variability seems low. The paper should

554	potentially compare the modeled Efire against something like GFED, and explain the ramifications of

555	any differences on their estimate of the role of managed vs unmanaged lands and the climate

556	sensitivity thereof.

557

558	The modeled _Efire_ in HN2017 and ORCHIDEE includes two components: deforestation-driven

559	emissions and emissions from fuel wood collection and usage. The latter part is not included in

560	GFED4s data. We compared the deforestation-driven component of _Efire_ with the deforestation fire

561	carbon emissions estimated by GFED4s. The latter covers only the period since 1997. As shown in

562	Figure R4, deforestation emissions by both HN2017 and ORCHIDEE closely follow the temporal

563	changes of deforestation area, which is expected because a fixed fraction of aboveground biomass

564	was partitioned as fire-driven fluxes in deforestation in both methods.

565

566	However, the IAVs of deforestation-driven emissions since 1997 by both HN2017 and ORCHIDEE are

567	lower than GFED4s. This mismatch is due to several reasons. First, the forest area changed as the

568	forcing data of HN2017 (used in ORCHIDEE) were based on the Forest Resource Assessment (FRA) by

569	FAO, which reports forest area changes every five years but not annually. Therefore, the smoothed

570	forest area loss in the forcing data contributes to the lower IAV in HN2017 and ORCHIDEE estimates.

571	We believe this is the major reason.

572

573	Second, deforestation fire emissions from GFED4s are not directly derived from satellite-based

574	annual forest loss, but based on a proxy calculated as the product of woody burned area and times of

575	burning during a given year. This approach likely captures better the IAV of fire-driven deforestation

576	emissions because it can reflect the potential lag between the times when a forest is cut and when

577	the cleared aboveground biomass is actually burned. In modern times forests can be cut down by

578	machines, or directly burned by fires. For those cut down by machines, burning of the cleared

579	biomass does not follow immediately but has to wait until suitable meteorological conditions occur.

580	It remains unclear how much is the fraction of the deforestation area actually made by fire*,* but this

581	fraction is likely higher in historical times and lower in modern days.

582

583	Third, GFED4s used a dynamic combustion loss fraction that translates the burned biomass into CO2

584	emissions, while in HN2017 and ORCHIDEE a fixed value was used. This might be the second major

585	reason that GFED4s shows higher IAV than the other two estimates.

586

587	To address all these issues, a dedicated module describing relevant fire processes in deforestation is

588	needed. This development is underway in the ORCHIDEE model, but was not yet ready for this study.

589

590	In summary, both HN2017 and ORCHIDEE results likely underestimated the IAV of fire-driven

591	deforestation emissions because 5-year smoothed deforestation area and static combustion loss

592	coefficients were used. This implies that the contribution of _ELUC_ to IAV of _Snet,_ and the temperature

593	sensitivity of _ELUC_ are also underestimated. We have briefly discussed these points in the revised

594	manuscript in lines 239-244.

595

596

597	**Figure R4. Deforestation-driven component of _Efire_ simulated by ORCHIDEE, HN2017, and the**

598	**deforestation fire carbon emissions estimated by GFED4s.** For comparison, the deforestation area

599	used in the model is also shown (the thick gray line on the right vertical axis). Except for the strong La

600	Nina years around 2000, the 5-year smooth emissions by GFED4s show similar IAV as HN2017 and

601	ORCHIDEE.

602

603	[6] As more minor comments, Fig. 2 should have labels on the color bars. Fig. 5 should have the flux

604	indicated on the y-axis, not in the upper left-hand corner of each panel.

605

606	Both figures (now Fig. 2 and Fig. 6 in the revised manuscript) have been changed according to the

607	reviewer’s suggestion.

608

609	[7] Figs 1-5 are relatively blurry, whereas Fig. 6 looks very crisp. Whatever graphics rendering was

610	used for Fig. 6 should be used throughout for the final publication.

611

612	Sometimes post-treatment of figures in Microsoft Powerpoint and pasting back in Microsoft Word

613	can result in blurry figures. We will provide high-quality pdf files for all figures to maximize their

614	clarity when the manuscript moves to final publication.

615

616	[8] Line 77 “is the composite term of these component fluxes” – could this be stated more simply as

617	“is the sum of these component fluxes”??

618

619	We changed the text as proposed by the reviewer.

620

621	[9] Line 84 What is “it” in the subject?

622

623	We changed this sentence to: “Bookkeeping models excel in ...”

624

625	[10] Line 100 “it’s necessary DGVM’s capabilities” => “it is necessary that the capabilities of DGVMs

626	be expanded”

627

628	We changed the texts as proposed by the reviewer.

629

630

631	**References:**

632

633	Doughty, R., Xiao, X., Wu, X., Zhang, Y., Bajgain, R., Zhou, Y., Qin, Y., Zou, Z., McCarthy, H., Friedman,

634	J., Wagle, P., Basara, J. and Steiner, J.: Responses of gross primary production of grasslands and

635	croplands under drought, pluvial, and irrigation conditions during 2010–2016, Oklahoma, USA,

636	Agricultural Water Management, 204, 47–59, doi:10.1016/j.agwat.2018.04.001, 2018.

637	Houghton, R. A. and Nassikas, A. A.: Global and regional fluxes of carbon from land use and land

638	cover change 1850–2015, Global Biogeochem. Cycles, 31(3), 2016GB005546,

639	doi:10.1002/2016GB005546, 2017.

640	Houghton, R. A. and Nassikas, A. A.: Negative emissions from stopping deforestation and forest

641	degradation, globally, Global change biology, 24(1), 350–359, 2018.

642	Hurtt, G. C., Frolking, S., Fearon, M. G., Moore, B., Shevliakova, E., Malyshev, S., Pacala, S. W. and

643	Houghton, R. A.: The underpinnings of land-use history: three centuries of global gridded land-use

644	transitions, wood-harvest activity, and resulting secondary lands, Global Change Biology, 12(7),

645	1208–1229, doi:10.1111/j.1365-2486.2006.01150.x, 2006.

646	Koch, A., Brierley, C., Maslin, M. M. and Lewis, S. L.: Earth system impacts of the European arrival and

647	Great Dying in the Americas after 1492, Quaternary Science Reviews, 207, 13–36,

648	doi:10.1016/j.quascirev.2018.12.004, 2019.

649	Mertz, O., Wadley, R. L., Nielsen, U., Bruun, T. B., Colfer, C. J. P., de Neergaard, A., Jepsen, M. R.,

650	Martinussen, T., Zhao, Q., Noweg, G. T. and Magid, J.: A fresh look at shifting cultivation: Fallow

651	length an uncertain indicator of productivity, Agricultural Systems, 96(1–3), 75–84,

652	doi:10.1016/j.agsy.2007.06.002, 2008.

653	Pan, Y., Birdsey, R. A., Fang, J., Houghton, R., Kauppi, P. E., Kurz, W. A., Phillips, O. L., Shvidenko, A.,

654	Lewis, S. L., Canadell, J. G., Ciais, P., Jackson, R. B., Pacala, S. W., McGuire, A. D., Piao, S., Rautiainen,

655	A., Sitch, S. and Hayes, D.: A Large and Persistent Carbon Sink in the World’s Forests, Science,

656	333(6045), 988–993, doi:10.1126/science.1201609, 2011.

657	Pugh, T. A. M., Lindeskog, M., Smith, B., Poulter, B., Arneth, A., Haverd, V. and Calle, L.: Role of forest

658	regrowth in global carbon sink dynamics, Proc Natl Acad Sci USA, 116(10), 4382,

659	doi:10.1073/pnas.1810512116, 2019.

660	Shevliakova, E., Pacala, S. W., Malyshev, S., Hurtt, G. C., Milly, P. C. D., Caspersen, J. P., Sentman, L. T.,

661	Fisk, J. P., Wirth, C. and Crevoisier, C.: Carbon cycling under 300 years of land use change: Importance

662	of the secondary vegetation sink, Global Biogeochem. Cycles, 23(2), GB2022,

663	doi:10.1029/2007GB003176, 2009.

664	Smith, P. C., Noblet-Ducoudré, N. D., Ciais, P., Peylin, P., Viovy, N., Meurdesoif, Y. and Bondeau, A.:

665	European-wide simulations of croplands using an improved terrestrial biosphere model: Phenology

666	and productivity, Journal of Geophysical Research: Biogeosciences, 115(G1),

667	doi:10.1029/2008JG000800, 2010.

668	Yang, X., Richardson, T. K. and Jain, A. K.: Contributions of secondary forest and nitrogen dynamics to

669	terrestrial carbon uptake, Biogeosciences, 7(10), 3041–3050, doi:10.5194/bg-7-3041-2010, 2010.

**Reviewer comments, second version:**

Reviewer #1 (Remarks to the Author):

My concerns were very thoroughly adressed in the main text of the manuscript and in the SI, and the revisions were explained and justified in detail in the response letter. In consider the manuscript ready for publication now.


Reviewer #2 (Remarks to the Author):

Yue et al., Contribution of land use on the interannual variability of the land carbon cycle (resubmitted version)

I appreciate the efforts done by the authors in revising the paper, and am particularly glad to see the revised method for accounting for land use. I agree that this may give some discrepancies with the bookkeeping method, but this is also because the bookkeeping method has not been designed to assess interannual variability caused by climatic drivers. It surprised me that the "unchanged" agricultural land contributes only little to IAV (Fig. S10), but I am glad that you have tested this and think that it is more correct to account for it in this way.

The alterations have improved the manuscript and I recommend it for publication in Nature Communications. I have a few minor comments to the current version of the manuscript that I would like to share with the authors:

- In the new text, reference is made to Fig. 3, 6a and 7a (l. 137, l. 140), while the analysis of these is only done further down in the text and it breaks the logical order of the figures. I would suggest to remove reference to the figures here and use these instances just to indicate that such comparisons are made later.
- l. 186: clarify that this refers to the distinction between intact and managed forest, not between temperate and boreal regions.
- l. 239: “However” can be removed.
- l. 348: “was” should read “were”
- l. 352: “Because 17% of global cropland was irrigated as of 2015”: Please add a reference for this.
- l. 616: “computing source demand” may not be entirely clear. Do you mean “computing resources”?


Reviewer #3 (Remarks to the Author):

The authors have provided an improved manuscript with a more detailed supplement that has adequately addressed my concerns, especially about model evaluation. It seemed that adequate responses were also provided to the concerns raised by the other two reviewers. I find the additions and changes to the text to be adequate and the manuscript is suitable for publication.

**Author rebuttal, second version:**

We thank for all the three reviewers’ efforts in reviewing our paper and for their appreciation of our revision efforts. Below please find out point-to-point for the further minor comments raised by #2 Reviewer.

Yue et al., Contribution of land use on the interannual variability of the land carbon cycle (resubmitted version)

I appreciate the efforts done by the authors in revising the paper, and am particularly glad to see the revised method for accounting for land use. I agree that this may give some discrepancies with the bookkeeping method, but this is also because the bookkeeping method has not been designed to assess interannual variability caused by climatic drivers. It surprised me that the "unchanged" agricultural land contributes only little to IAV (Fig. S10), but I am glad that you have tested this and think that it is more correct to account for it in this way.

The alterations have improved the manuscript and I recommend it for publication in Nature Communications. I have a few minor comments to the current version of the manuscript that I would like to share with the authors:

- In the new text, reference is made to Fig. 3, 6a and 7a (l. 137, l. 140), while the analysis of these is only done further down in the text and it breaks the logical order of the figures. I would suggest to remove reference to the figures here and use these instances just to indicate that such comparisons are made later.

Following the reviewer’s suggestion, we removed these figure citations.

- l. 186: clarify that this refers to the distinction between intact and managed forest, not between temperate and boreal regions.

We changed to this sentence to: because distinctions were not made between these two forest types in the latter.

- l. 239: “However” can be removed.
This is done.

- l. 348: “was” should read “were”
Change has been made.

- l. 352: “Because 17% of global cropland was irrigated as of 2015”: Please add a reference for this.

We added the reference of the HYDE3.2 data by Klein Goldewijk et al. (2017)

- l. 616: “computing source demand” may not be entirely clear. Do you mean “computing resources”?

Sorry for the typo. We indeed meant “computing resources” and this has been corrected.

**Reviewer comments, third version:**

**Author rebuttal, third version:**
